# Stereoselective Synthesis of (*R*)-all-*trans*-13,14-Dihydroretinol and -Retinoic Acid

**DOI:** 10.1021/acs.joc.4c03173

**Published:** 2025-02-20

**Authors:** Paul Wienecke, Adriaan J. Minnaard

**Affiliations:** Stratingh Institute for Chemistry, University of Groningen, Nijenborgh 7, 9747 AG, Groningen, The Netherlands

## Abstract

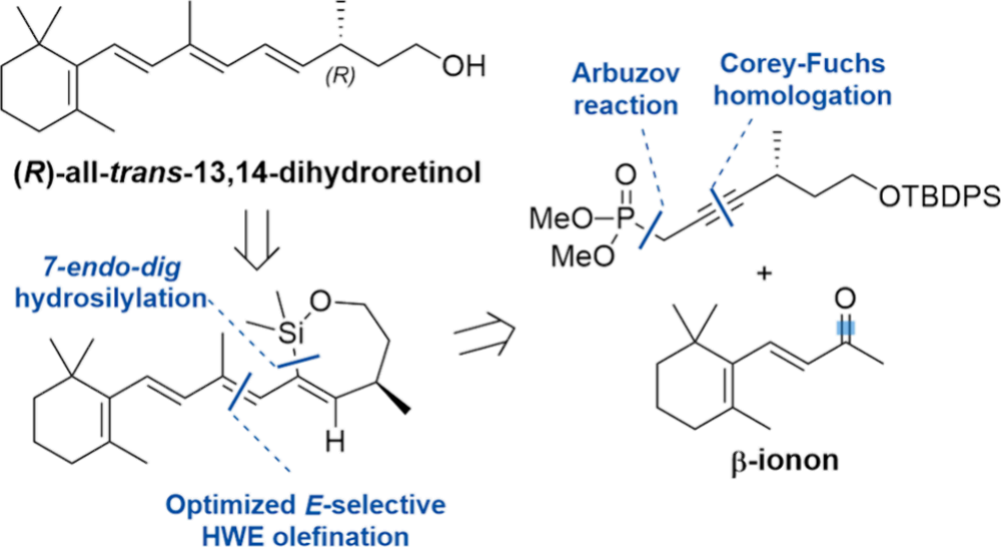

Vitamin A (or all-*trans*-retinol) metabolites are involved in a wide range of cellular processes. However, the investigation of their biological role is hampered due to their very limited availability. Herein we report a stereoselective total synthesis of the vitamin A metabolites (*R*)-all-*trans*-13,14-dihydroretinol and -retinoic acid, applying an *E*-selective HWE olefination and a Ru(II) catalyzed intramolecular 7-*endo*-*dig* hydrosilylation as the key steps.

All-*trans*-retinol (**1**, Scheme 1), or vitamin A, is a fat-soluble, essential nutrient that humans need to obtain from their plant- or animal-based diet.^1^ In a broader sense, the term vitamin A also includes structurally related compounds, for instance, retinol metabolites and retinyl esters. This “vitamin A” plays important roles in a wide range of physiological processes, such as vision, cell differentiation, immune response, central nervous system development and embryogenesis.^2^ However, the very limited access to vitamin A metabolites hampers their investigation, leading to their poorly understood intertwinement with human body functions.^3^ A common metabolic pathway of all-*trans*-retinol (**1**) in vertebrates is the net hydrogenation to (*R*)-all-*trans*-13,14-dihydroretinol (**2**, Scheme 1) by the oxidoreductase retinol saturase (RetSat).^4−6^ This enzyme’s known involvement in adipocyte differentiation, liver metabolism, macrophage function and reactive oxygen species formation as well as its links to diabetes and tumor development underlines its central physiological role, but deeper understanding is lacking.^7^ The metabolic fate of (*R*)-all-*trans*-13,14-dihydroretinol (**2**) is similar to that of all-*trans*-retinol (**1**) and depends on the same enzymes (Scheme 1): First, **2** is oxidized to (*R*)-all-*trans*-13,14-dihydroretinal (**4**) by broad-spectrum dehydrogenases, followed by irreversible oxidation to (*R*)-all-*trans*-13,14-dihydroretinoic acid (**6**) by a retinal dehydrogenase.^8^

**Scheme 1:**
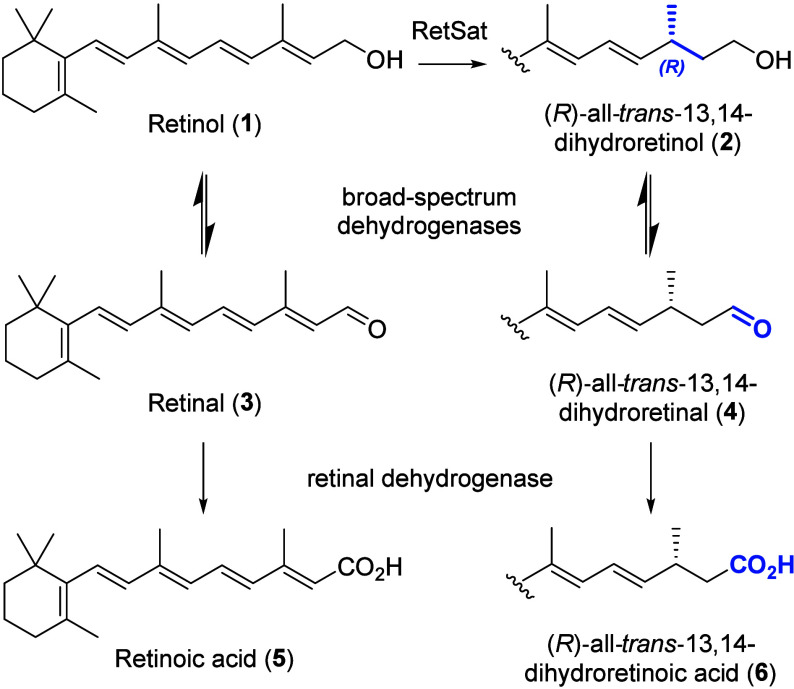
Metabolism of Retinol (**1**) and (*R*)-all-*trans*-13,14-Dihydroretinol (**2**)^8^

(*R*)-all-*trans*-13,14-dihydroretinol (**2**) and its corresponding carboxylic acid **6** are potent and selective activators of the retinoic acid receptor *in vitro*, likewise as their all-*trans*-retinol counterparts **1** and **5**.^8^ However, the potency of **2** and **6** declines tremendously *in vivo*, probably below a physiological relevant threshold. Although they are metabolically more stable, a less efficient protein-mediated nuclear transport seems to account for this phenomenon.^9^ To date, possible extranuclear targets have not been investigated and the biological functions of (*R*)-all-*trans*-13,14-dihydroretinol (**2**) and its metabolites remain cryptic.^10^ The restricted access to these compounds poses a serious bottleneck for activity studies, for whom substantial amounts are needed. The single published synthesis allowed access and enabled the assignment of the absolute configuration,^6^ but the requirement for step-intensive sequences, such as diastereoselective formation of the stereocenter, and high excesses of CrCl_2_ and TlOH limits its applicability.

We aimed at a stereoselective total synthesis of (*R*)-all-*trans*-13,14-dihydroretinol (**2**) and the corresponding acid **6** to facilitate the elucidation of their functions in the human body. The double bond configurations in retinol and its derivatives play an important role in their bioactivities.^3^ Therefore, in planning the synthesis, we emphasized on eminently *trans*-selective alkene formations. Furthermore, the sensitivity of the conjugated alkenes toward oxidants, acids, heat and light had to be taken into account. In our retrosynthetic analysis, we envisioned (*R*)-all-*trans*-13,14-dihydroretinol (**2**) to be obtained from a stereoretentive desilylation after an alcohol-directed, intramolecular hydrosilylation of trien-yne **8** (Scheme 1). *7-endo-dig* ring-closure to oxasilacycloheptene **7** would lead to an *E*-specific formation of the 11,12-double bond. In contrast, *6-exo-dig* cyclization would also give the undesired *Z-*isomer.^11^ Considering the successful application of stereoselective olefinations in the synthesis of vitamin A derivatives,^6,12,13^ trien-yne **8** was planned to be accessed by an *E*-selective Horner-Wadsworth-Emmons (HWE) reaction of propargylic phosphonate **10** with β-ionone (**9**). We chose to synthesize phosphonate **10** from commercially available enantiopure *R*-(+)-methylsuccinic acid (**11**, the *S*-enantiomer is available as well), featuring the natural absolute configuration, via an Arbuzov reaction and a Corey-Fuchs homologation.

The synthesis of phosphonate **10** began with the reduction of (*R*)-(+)-methylsuccinic acid (**11**) to the diol by LiAlH_4_ (Scheme 2). Subsequent low-temperature TBDPS-silylation^14^ furnished the monoprotected diol **12** (Scheme 3) with good site-selectivity. Subsequent Parikh–Doering oxidation of the hydroxyl group gave aldehyde **13** in a very good yield. Following a Corey-Fuchs homologation approach, aldehyde **13** was first transformed into the corresponding dibromoalkene. Treatment with *n*BuLi and trapping the resulting alkynyllithium intermediate with paraformaldehyde, yielded efficiently propargyl alcohol **14** as the precursor for phosphonate **10**.

**Scheme 2:**
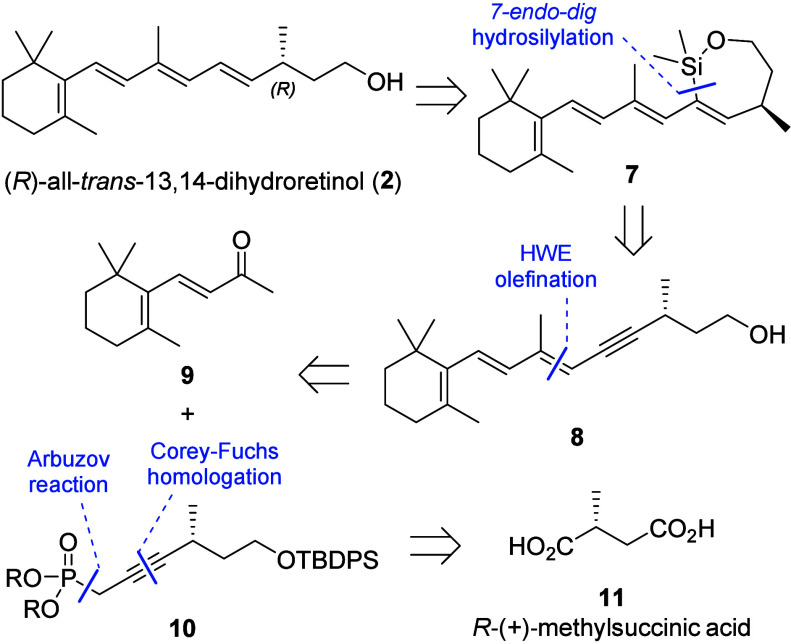
Retrosynthetic Analysis of (*R*)-all-*trans*-13,14-Dihydroretinol (**2**)

**Scheme 3:**
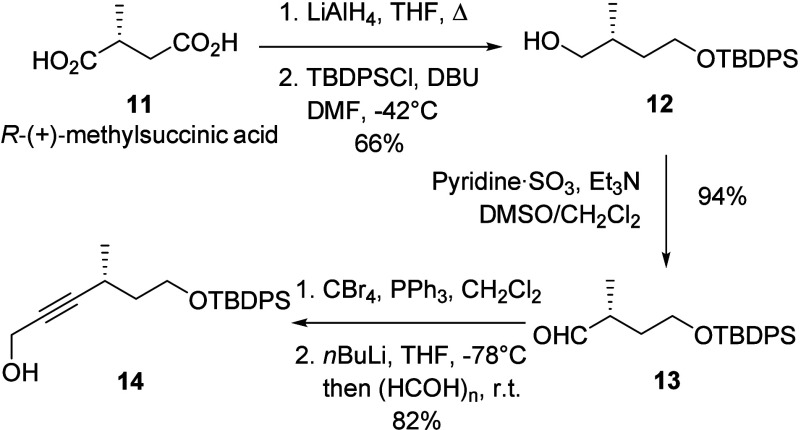
Synthesis of Propargyl Alcohol **14** from (*R*)-(+)-Methylsuccinic Acid (**11**)

In literature, HWE olefinations of β-ionone with propargylic phosphonates led to considerable amounts of *Z*-alkene byproducts.^15−17^ To improve *E*-selectivity of the envisioned olefination, we performed model optimization studies with readily available TMS-protected propargyl phosphonate **15**, giving trien-yne **16** in good to very good yields and short reaction times (Table 1). It is worth mentioning that the Julia-Kocienski olefination reagents **17** and **18** did not give any conversion. As Julia-Kocienski olefinations of β-ionone (**9**) with nonstabilized aliphatic α-sulfonyl anions are known,^18^ it is likely that the stabilized propargylic anions exhibit insufficient reactivity for this transformation. An initial screening of bases for the deprotonation of the phosphonate (entries 1–4) revealed that NaHMDS gave the highest *E*-selectivity in comparison to the corresponding Li and K bases. Employing *n*BuLi instead of LiHMDS did not have a significant impact on the selectivity. Weaker bases such as NaOCH(CF_3_)_2_, Ba(OH)_2_, DBU/LiBr or Triton B gave no or sluggish conversion (data not shown). Typical for stabilized α-phosphonate anions, is that the initial addition to the carbonyl group favors a *Z*-transition state, while the subsequent rate-determining oxyanion cyclization favors an *E*-transition state. Reaction parameters such as the selection of the countercation influence energy levels of transition states and reversibility, and therefore, also *E*/*Z* selectivity.^19−22^ Changing the reaction solvent to the less coordinating toluene considerably improved the *E*/*Z* ratio (entry 5). The same was observed for Et_2_O, although less pronounced (entry 6). DME and MTBE gave lower *E*/*Z* ratios (entries 7 and 8), comparable to THF (entry 3). Further enhancing cation solvation by using DMPU as the cosolvent increased the formation of the *Z*-alkene (entry 9). By lowering the reaction temperature to −78 °C, a very good *E*/*Z* ratio of 9:1 was achieved (entry 10). Unfortunately, and unexpectedly, employing a smaller excess of the base and phosphonate **15** gave a diminished stereoselectivity (entry 11). Larger substituents on the phosphonate had no positive effect (entry 12 and 13). While BINOL-based phosphonates occasionally give high *Z*-selectivity in HWE olefinations,^23^ this preference was not observed in our case (entry 14).

**Table 1:**
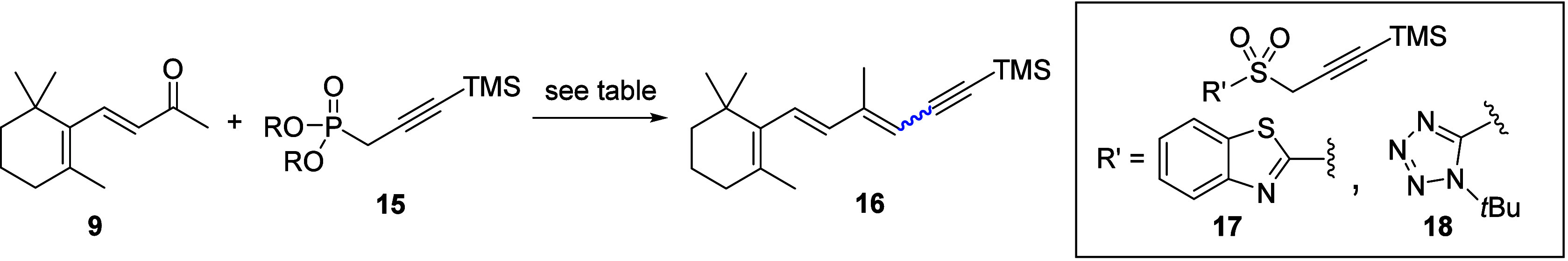
Conditions Screening for a Model HWE Olefination of β-Ionone (9) with Phosphonates 15^a^

Entry	R	Base	Solvent	Temp	Conv. (%)^b^	*E*/*Z*
1	Et	LiHMDS	THF	–10 °C	87 (84)^c^	69:31
2	Et	*n*BuLi	THF	–10 °C	79%	68:32
3	Et	NaHMDS	THF	–10 °C	88%	72:28
4	Et	KHMDS	THF	–10 °C	85%	67:33
5	Et	NaHMDS	toluene	–10 °C	90%	84:16
6	Et	NaHMDS	Et_2_O	–10 °C	85%	80:20
7	Et	NaHMDS	DME	–10 °C	85%	69:31
8	Et	NaHMDS	MTBE	–10 °C	80%	70:30
9	Et	NaHMDS	THF/DMPU (1:1)	–10 °C	86%	64:36
**10**	***Et***	***NaHMDS***	***toluene***	***-78 °C***	***88%***	***90:10***
11	Et	NaHMDS^d^	toluene	–78 °C	77%	69:31
12	*i*Pr	NaHMDS	toluene	–78 °C	77%	88:12
13	Bn	NaHMDS	toluene	–78 °C	80%	84:16
14	BINOL	KHMDS	THF	–78 °C	62%	77:23

a **9** (0.1 mmol), NaHMDS (2.0 equiv), **15** (2.0 equiv), solvent (0.1 M); 0.5 h stirring for phosphonate deprotonation before addition of **9**, 0.5 h further stirring.

b As monitored by NMR using 1,3,5-trimethoxybenzene as internal standard.

c Isolated yield.

d 1.2 equiv of base and **15**, 4 h reaction time.

As larger phosphonate substituents did not show a positive influence on the *E*/*Z*-selectivity, we aimed at transforming propargyl alcohol **14** into the corresponding dimethylphosphonate **19**. Synthesis of the propargylic bromide via an Appel reaction set the stage for phosphonylation by an Arbuzov reaction, effected by refluxing in trimethyl phosphite (Scheme 4). This method furnished phosphonate **19** on multigram-scale in an excellent yield.

**Scheme 4:**
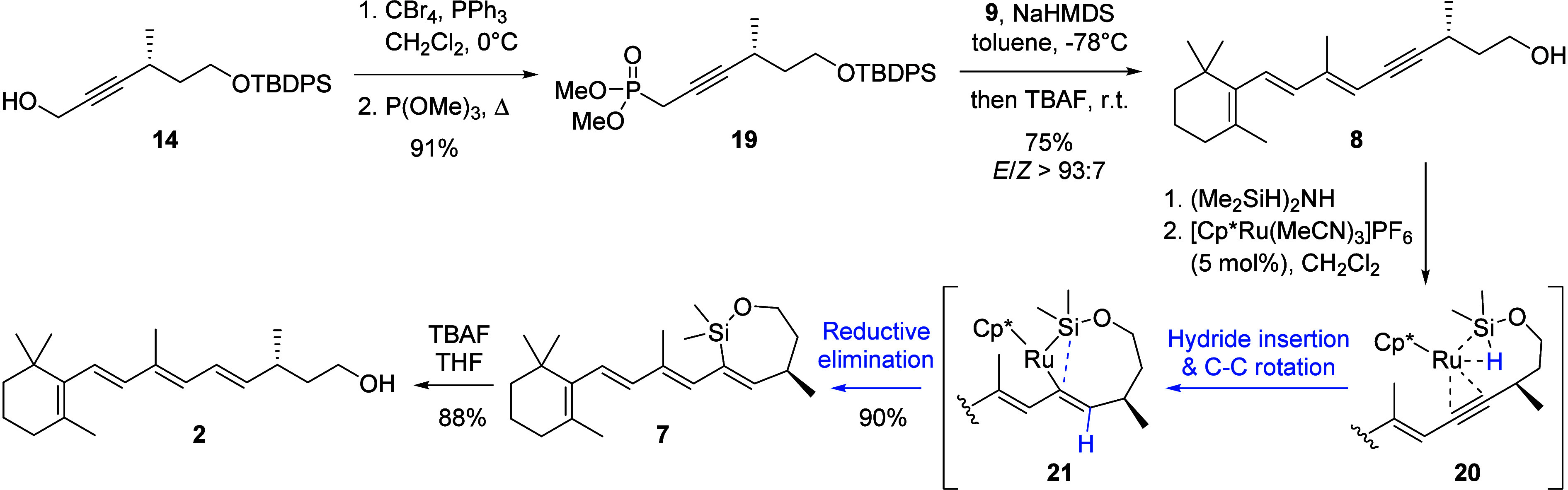
Synthesis of all-*trans*-13,14-Dihydroretinol (**2**) from Propargylic Alcohol **14** The MeCN ligands in the simplified transition states **20** and **21** are omitted for clarity.

To our delight, transferring the optimized HWE olefination conditions to the synthesis of trien-yne alcohol **8** resulted in a satisfying yield and very good *E*-selectivity (*E*/*Z* > 93:7) after one-pot desilylation. Trien-yne alcohol **8** was treated with neat tetramethyldisilazane resulting in the corresponding dimethylsilyl ether, serving as the substrate for a *7-endo-dig* hydrosilylation. The course of intramolecular alkyne hydrosilylations depends on the employed catalyst: While Pt and most Ru catalysts effect *exo* cyclizations,^11,24^ the cationic Ru complex [Cp*Ru(MeCN)_3_]PF_6_ has been reported to give *endo* cyclization products with high selectivity.^25,26^ Mechanistically, DFT calculations^27^ indicate an *endo* transition state similar to **20**. Subsequent hydride insertion is accompanied by C–C bond rotation, placing the hydride *anti* to the silyl group as in simplified transition state **21**, thereby establishing the observed *trans* selectivity. Reductive elimination leads to oxasilacycloheptene **7** with 90% yield from trien-yne alcohol **8**. Finally, desilylation of **7** by treatment with TBAF yielded (*R*)-all-*trans*-13,14-dihydroretinol (**2**). The analytical data matched to those previously reported. This synthesis route enables an unprecedented efficient preparation of (*R*)-all-*trans*-13,14-dihydroretinol (**2**), omitting tedious stereocenter formation and carbon skeleton assembly as in the previous published synthesis.^6^

Having established synthetic access to (*R*)-all-*trans*-13,14-dihydroretinol (**2**), we explored its transformation into its main oxidized metabolites. Careful selection of oxidation conditions was required here to keep the sensitive tetraene unit intact. DMP-oxidation of **2** gave (*R*)-all-*trans*-13,14-dihydroretinal (**4**) successfully (Scheme 5). Treatment with *in situ* formed Ag(I) oxide, known to not affect alkenes,^28^ furnished (*R*)-all-*trans*-13,14-dihydroretinoic acid (**6**), showing gratifying compatibility.

**Scheme 5:**
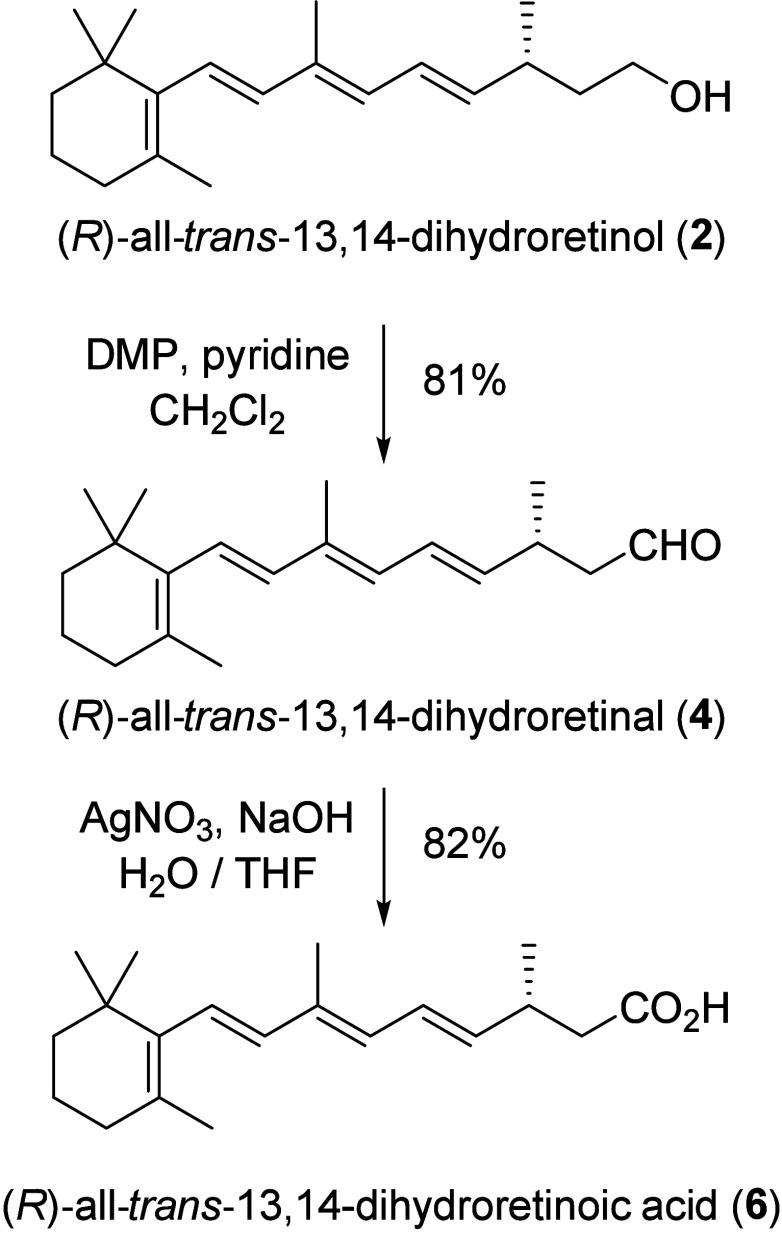
Transformation of (R)-all-*trans*-13,14-Dihydroretinol (**2**) to the Related Metabolites **4** and **6**

In conclusion, we developed an efficient and stereoselective total synthesis of the vitamin A metabolite (*R*)-all-*trans*-13,14-dihydroretinol (**2**), with an optimized *E*-selective HWE olefination and a Ru(II) catalyzed intramolecular 7-*endo*-*dig* hydrosilylation as the key steps. Furthermore, we have found compatible conditions for stepwise oxidation to (*R*)-all-*trans*-13,14-dihydroretinoic acid (**6**). We are convinced that this novel access to these molecules will facilitate the elucidation of their biological function in future studies.

## Experimental Section

### General Experimental Information

All reactions were carried out under a nitrogen atmosphere using standard Schlenk techniques. Reaction temperatures refer to the temperature of the heating mantle or cooling bath. Anhydrous solvents (MTBE, CH_2_Cl_2_, THF, toluene) were taken from an MBraun solvent purification system (SPS 800). Other anhydrous solvents were purchased from Sigma-Aldrich or Fisher Scientific and were used without further purification. Aqueous solutions are saturated if not mentioned otherwise. TLC analysis was performed on silica gel 60/Kieselguhr F254, 0.25 mm (Merck). Compounds were visualized using 254 nm UV light followed by KMnO_4_ stain. ^1^H, ^13^C{^1^H} and ^31^P NMR spectra were recorded on an Agilent 400 NMR spectrometer at 400, 101, and 162 MHz, respectively, using CDCl_3_ as the solvent. Chemical shifts are reported in ppm with the solvent resonance as the internal standard (for CDCl_3_: δ 7.26 ppm for ^1^H, δ 77.16 ppm for ^13^C). Data are reported as follows: chemical shifts (δ), multiplicity (s = singlet, d = doublet, t = triplet, q = quartet, sept = septet, b = broad, m = multiplet), coupling constant *J* (Hz), and integration value. High resolution mass spectra (HRMS) were recorded on a Thermo Scientific Orbitrap Exploris 480 mass spectrometer with electrospray ionization (ESI) or atmospheric pressure chemical ionization (APCI) in positive mode. Specific rotations were determined with a Schmidt + Haensch Polartronic MH8 polarimeter in a 100 mm path-length cell.

### Synthetic Procedures and Analytical Data

#### (R)-all-trans-13,14-Dihydroretinol (**2**)

To oxasilacycloheptene **7** (590 mg, 1.7 mmol, 1.0 equiv) in anhydrous THF (20 mL) at 0 °C was added TBAF (1 M in THF, 2.6 mL, 2.6 mmol, 1.5 equiv) and the reaction mixture was stirred for 2 h at r.t. Aqueous NH_4_Cl (20 mL) was added and the aqueous layer was extracted with Et_2_O (3 × 20 mL). The combined organic layers were washed with brine (30 mL) and dried over Na_2_SO_4_. Purification by flash column chromatography (pentane/Et_2_O = 8:2) gave (*R*)-all-*trans*-13,14-dihydroretinol **2** as a faint yellow oil (435 mg, 1.5 mmol, 88%).

TLC: *R*_f_ = 0.45 (pentane/Et_2_O = 6:4). ^**1**^H NMR (CDCl_3_, 400 MHz): δ = 6.41 (dd, *J* = 14.9, 11.1 Hz, 1 H), 6.11 (d, *J* = 16.2 Hz, 1 H), 6.04 (d, *J* = 16.2 Hz, 1 H), 5.99 (d, *J* = 11.1 Hz, 1 H), 5.60 (dd, *J* = 15.0, 8.4 Hz, 1 H), 3.70–3.61 (m, 2 H), 2.41 (sept, *J* = 7.1 Hz, 1 H), 2.00 (t, *J* = 6.8 Hz, 2 H), 1.90 (s, 3 H), 1.69 (s, 3 H), 1.65–1.55 (m, 4 H), 1.49–1.41 (m, 2 H), 1.06 (d, *J* = 6.7 Hz, 3 H), 1.01 (s, 6 H) ppm. ^13^C{^1^H} NMR (CDCl_3_, 101 MHz): 140.1, 138.0, 137.9, 134.4, 129.7, 129.0, 126.3, 125.8, 61.5, 40.0, 39.7, 34.5, 34.4, 33.1, 29.1, 21.8, 21.1, 19.4, 12.7 ppm. HRMS (ESI) *m*/*z* for [M + H]^+^ calculated for C_20_H_33_O 289.2526; found 289.2525. [α]_D_^20^ = −52 (*c* 0.1, CHCl_3_).

#### (R)-all-trans-13,14-Dihydroretinal (**4**)

To (*R*)-all-*trans*-13,14-DHR 2 (40 mg, 0.14 mmol, 1.0 equiv) and pyridine (11 μL, 0.14 mmol, 1.0 equiv) in anhydrous CH_2_Cl_2_ (2.5 mL) at 0 °C was added DMP (88 mg, 0.21 mmol, 1.5 equiv) and the reaction mixture was stirred for 1 h at r.t.. Direct purification by flash column chromatography (pentane/Et_2_O = 98:2) gave (*R*)-all-*trans*-13,14-dihydroretinal 4 as a faint yellow oil (32 mg, 0.11 mmol, 81%).

TLC: *R*_f_ = 0.50 (pentane/Et_2_O = 95:5). ^1^H NMR (CDCl_3_, 400 MHz): δ = 9.72 (b, 1 H), 6.41 (dd, *J* = 14.9, 11.2 Hz, 1 H), 6.11 (d, *J* = 16.1 Hz, 1 H), 6.02 (d, *J* = 16.1 Hz, 1 H), 5.96 (d, *J* = 11.0 Hz, 1 H), 5.63 (dd, *J* = 15.0, 7.6 Hz, 1 H), 2.86 (sept, *J* = 6.9 Hz, 1 H), 2.47 (ddd, *J* = 16.3, 7.0, 1.9 Hz, 1 H), 2.39 (ddd, *J* = 16.3, 6.9, 2.1 Hz, 1 H), 1.98 (t, *J* = 6.0 Hz, 2 H), 1.88 (s, 3 H), 1.67 (s, 3 H), 1.63–1.53 (m, 4 H), 1.47–1.40 (m, 2 H), 1.10 (d, *J* = 6.8 Hz, 3 H), 0.99 (s, 6 H) ppm. ^13^C{^1^H} NMR (CDCl_3_, 101 MHz): 202.4, 138.0, 137.8, 137.7, 135.2, 129.3, 129.1, 126.7, 126.2, 50.6, 39.7, 34.4, 33.1, 32.2, 29.1, 21.8, 20.7, 19.4, 12.7 ppm. HRMS (ESI) *m*/*z* for [M + H]^+^ calculated for C_20_H_31_O 287.2369; found 287.2372. [α]_D_^20^ = −58 (*c* 0.1, CHCl_3_).

#### (R)-all-trans-13,14-Dihydroretinoic Acid (**6**)

AgNO_3_ (41 mg, 0.24 mmol, 2.3 equiv) in water (2 mL) was added dropwise to NaOH (21 mg, 0.52 mmol, 5 equiv) in water (2 mL) at 0 °C. After stirring for 30 min in the dark, (*R*)-all-*trans*-13,14-dihydroretinal (**4**, 30 mg, 0.10 mmol, 1.0 equiv) in THF (0.5 mL) was added dropwise, the cooling bath was removed and stirring was continued for 12 h in the dark. The reaction mixture was subsequently poured into 0.5 M aqueous HCl (10 mL) and the aqueous layer was extracted with EtOAc (3 × 10 mL). The combined organic layers were dried over Na_2_SO_4_. Purification by flash column chromatography (CH_2_Cl_2_/MeOH = 95:5) gave (*R*)-all-*trans*-13,14-dihydroretinoic acid **6** as a faint yellow oil (26 mg, 86 μmol, 82%).

TLC: *R*_f_ = 0.48 (CH_2_Cl_2_/MeOH = 9:1). ^**1**^H NMR (CDCl_3_, 400 MHz): δ = 6.43 (dd, *J* = 14.9, 11.2 Hz, 1 H), 6.11 (d, *J* = 15.7 Hz, 1 H), 6.02 (d, *J* = 16.1 Hz, 1 H), 5.96 (d, *J* = 11.1 Hz, 1 H), 5.63 (dd, *J* = 15.0, 7.6 Hz, 1 H), 2.84–2.70 (m, 1 H), 2.45–2.25 (m, 2 H), 1.98 (t, *J* = 6.0 Hz, 2 H), 1.88 (s, 3 H), 1.67 (s, 3 H), 1.63–1.53 (m, 2 H), 1.47–1.40 (m, 2 H), 1.10 (d, *J* = 6.7 Hz, 3 H), 0.99 (s, 6 H) ppm. ^13^C{^1^H} NMR (CDCl_3_, 101 MHz): 178.1, 138.0, 137.8, 137.7, 135.0, 129.5, 129.1, 126.5, 126.1, 41.5, 39.7, 34.4, 34.0, 33.1, 29.1, 21.8, 20.4, 19.4, 12.7 ppm. HRMS (ESI) *m*/*z* for [M + H]^+^ calculated for C_20_H_31_O_2_ 303.2319; found 303.2318. [α]_D_^20^ = −46 (*c* 0.1, CHCl_3_).

#### (R)-2,2,5-Trimethyl-3-((1E,3E)-2-methyl-4-(2,6,6-trimethylcyclohex-1-en-1-yl)buta-1,3-dien-1-yl)-2,5,6,7-tetrahydro-1,2-oxasilepine (**7**)

Trien-yne alcohol **8** (667 mg, 2.3 mmol, 1.0 equiv) was dissolved in 1,1,3,3-tetramethyldisilazane (2.1 mL, 11.6 mmol, 5.0 equiv) and stirred for 18 h at r.t. Most of the solvent was evaporated *in vacuo* (approximately 4 mbar, 50 °C sand bath temperature) to give the crude dimethylsilyl ether that was taken to the next step without further purification.

The crude dimethylsilyl ether was dissolved in anhydrous CH_2_Cl_2_ (10 mL), cooled to 0 °C and Cp*Ru(MeCN)_4_PF_6_ (59 mg, 116 μmol, 5 mol %) was added. After stirring for 4 h at r.t., the reaction mixture was diluted with pentane (10 mL) and filtered over a short plug of florisil with the aid of pentane/Et_2_O (98:2). Drying the filtrate *in vacuo* yielded sufficiently pure oxasilacycloheptene **7** (720 mg, 2.1 mmol, 90%) as a yellow oil.

TLC: *R*_f_ = 0.40 (pentane/Et_2_O = 98:2). ^1^H NMR (CDCl_3_, 400 MHz): δ = 6.16–5.83 (m, 4 H), 3.98–3.78 (m, 2 H), 2.80–2.60 (m, 1 H), 1.98 (t, *J* = 6.0 Hz, 2 H), 1.95–1.88 (m, 1 H), 1.82 (s, 3 H), 1.69 (s, 3 H), 1.64–1.51 (m, 3 H), 1.48–1.40 (m, 2 H), 1.13 (d, *J* = 7.1 Hz, 3 H), 1.00 (s, 3 H) ppm. ^13^C{^1^H} NMR (CDCl_3_, 101 MHz): 150.8, 139.5, 138.3, 138.0, 134.5, 132.3, 128.7, 125.5, 62.3, 39.7, 38.2, 34.4, 33.3, 33.1, 29.1, 29.1, 22.2, 21.8, 19.4, 13.7, −0.6, −0.9 ppm. HRMS (APCI) *m*/*z* for [M + H]^+^ calculated for C_22_H_37_OSi 345.2608; found 345.2611.

#### (R,6E,8E)-3,7-Dimethyl-9-(2,6,6-trimethylcyclohex-1-en-1-yl)nona-6,8-dien-4-yn-1-ol (**8**)

NaHMDS (2 M in THF, 3.1 mL, 6.2 mmol, 2.0 equiv) was added to phosphonate **19** (2.86 g, 6.2 mmol, 2.0 equiv) in anhydrous toluene (24 mL) at −78 °C and the mixture was stirred for 30 min. β-ionone (635 μL, 3.1 mmol, 1.0 equiv) in precooled anhydrous toluene (6 mL) was added dropwise and the reaction mixture was stirred for 1 h. Afterward, TBAF (1 M in THF, 9.4 mL, 9.4 mmol, 3.0 equiv) was added and after warming to r.t., stirring was continued for 5 h. Aqueous NH_4_Cl (20 mL) was added and the aqueous layer was extracted with Et_2_O (3 × 30 mL). The combined organic layers were washed with brine (50 mL) and dried over Na_2_SO_4_. Purification by flash column chromatography (pentane/Et_2_O = 7:3) gave the desired trien-yne alcohol **8** as a yellow oil (667 mg, 2.3 mmol, 75%).

TLC: *R*_f_ = 0.34 (pentane/Et_2_O = 8:2). ^**1**^H NMR (CDCl_3_, 400 MHz): δ = 6.17 (d, *J* = 15.8 Hz, 1 H), 6.04 (d, *J* = 16.1 Hz, 1 H), 5.38 (s, 1 H), 3.87–3.75 (m, 2 H), 2.87–2.75 (m, 1 H), 2.03–1.95 (m, 2 H), 2.00 (s, 3 H), 1.79–1.68 (m, 3 H), 1.66 (s, 3 H), 1.62–1.54 (m, 2 H), 1.47–1.40 (m, 2 H), 1.24 (d, *J* = 6.9 Hz, 3 H), 0.98 (s, 3 H) ppm. ^13^C{^1^H}-NMR (CDCl_3_, 101 MHz): 146.5, 137.6, 135.7, 129.8, 129.0, 108.8, 100.7, 80.2, 61.4, 39.8, 39.6, 34.3, 33.1, 29.0, 24.1, 21.8, 21.6, 19.4, 15.1 ppm. HRMS (APCI) *m*/*z* for [M + H]^+^ calculated for C_20_H_31_O 287.2369; found 287.2370. [α]_D_^20^ = −38 (*c* 0.1, CHCl_3_).

#### (R)-4-((*tert*-Butyldiphenylsilyl)oxy)-2-methylbutan-1-ol (**12**)

LiAlH_4_ (2.56 g, 68.1 mmol, 3.0 equiv) was added portionwise to *R*-(+)-methylsuccinic acid (**11**, 3.00 g, 22.7 mmol, 1.0 equiv) in anhydrous THF (80 mL) at 0 °C. The mixture was stirred for 1 h at r.t. and then for 15 h at 60 °C by heating with a sand bath. After cooling to r.t., Rochelle salt (38.5 g, 136 mmol, 6 equiv) and water (6 mL) were added and stirring was continued overnight. The mixture was filtered over a short plug of Celite with the aid of THF. The filtrate was dried *in vacuo* and the crude diol was taken to the next step without further purification.

The crude diol was dissolved in anhydrous DMF (90 mL), cooled to −40 °C and DBU (5.1 mL, 34.1 mmol, 1.5 equiv) was added. Then, TBDPSCl (5.9 mL, 22.7 mmol, 1.0 equiv) was added over 2 h by syringe pump. Stirring was continued for 8 h before quenching the reaction by addition of aqueous NaHCO_3_ (100 mL). The aqueous layer was extracted with Et_2_O (3 × 100 mL) and the combined organic layers were washed with brine (200 mL). Drying over Na_2_SO_4_ and purification by flash column chromatography (pentane/Et_2_O = 8:2) afforded the desired alcohol **12** (5.14 g, 15.0 mmol, 66% over two steps) as a colorless oil. The analytical data matched to those previously reported.^29^

#### (R)-4-((*tert*-Butyldiphenylsilyl)oxy)-2-methylbutanal (**13**)

Et_3_N (8.3 mL, 60.0 mmol, 4.0 equiv) and pyridine-SO_3_ (4.78 g, 30.0 mmol, 2.0 equiv) were added to alcohol **12** (5.14 g, 15.0 mmol, 1.0 equiv) in anhydrous CH_2_Cl_2_ (120 mL) and anhydrous DMSO (30 mL) at 0 °C. After stirring for 1 h at r.t., aqueous NH_4_Cl (120 mL) was added and the aqueous layer was extracted with CH_2_Cl_2_ (3 × 100 mL). The combined organic layers were washed with brine (200 mL) and dried over Na_2_SO_4_. Purification by flash column chromatography (pentane/Et_2_O = 98:2) yielded the desired aldehyde **13** as a colorless oil (4.79 g, 14.1 mmol, 94%). The analytical data matched to those previously reported.^30^

#### (R)-6-((*tert*-Butyldiphenylsilyl)oxy)-4-methylhex-2-yn-1-ol (**14**)

CBr_4_ (7.79 g, 23.5 mmol, 2.0 equiv) was added portionwise to aldehyde **13** (4.00 g, 11.7 mmol, 1.0 equiv) and PPh_3_ (12.3 g, 47.0 mmol, 4.0 equiv) in CH_2_Cl_2_ (70 mL) at 0 °C. After stirring for 1 h at r.t., most of the solvent was evaporated and the residue diluted with pentane (150 mL). Filtration over a short plug of SiO_2_ with the aid of pentane and drying of the filtrate *in vacuo* gave the desired dibromoalkene that was taken to the next step without further purification.

To the crude dibromoalkene in anhydrous THF (50 mL) at −78 °C was added *n*BuLi (1.6 M in hexane, 16.2 mL, 25.8 mmol, 2.2 equiv) dropwise. The reaction mixture was stirred for 1.5 h and then warmed to 0 °C. After stirring for 30 min, paraformaldehyde (0.81 g, 27.0 mmol, 2.3 equiv) was added, the ice-bath was removed and stirring was continued for 15 h. Aqueous NH_4_Cl (50 mL) was added and the aqueous layer was extracted with Et_2_O (3 × 50 mL). The combined organic layers were dried over Na_2_SO_4_. Purification by flash column chromatography (pentane/Et_2_O = 8:2) yielded the desired propargyl alcohol **14** as a colorless oil (3.52 g, 9.6 mmol, 82% over two steps).

TLC: *R*_f_ = 0.37 (pentane/Et_2_O = 8:2). ^1^H NMR (CDCl_3_, 400 MHz): δ = 7.74–7.64 (m, 4 H), 7.47–7.35 (m, 6 H), 4.19 (d, *J* = 2.0 Hz, 2 H), 3.86–3.72 (m, 2 H), 2.82–2.72 (m, 1 H), 1.77–1.60 (m, 2 H), 1.43 (s, 1 H), 1.17 (d, *J* = 7.0 Hz, 3 H), 1.06 (s, 9 H) ppm. ^13^C{^1^H} NMR (CDCl_3_, 101 MHz): δ = 135.8*, 135.7*, 134.02*, 133.97*, 129.7, 127.73*, 127.71*, 90.5, 78.8, 61.7, 51.5, 39.6, 27.0, 22.5, 21.0, 19.3 ppm. HRMS (APCI) *m*/*z* for [M + H]^+^ calculated for C_23_H_31_O_2_Si 367.2088; found 367.2088. [α]_D_^20^ = −40 (*c* 0.1, CHCl_3_).

*1:1 splitted ^13^C NMR signals are partially observed for aromatic carbons in the TBDPS substituent. This indicates hindered rotation of the silyl ether, leading to rotamers.

#### Dibenzyl (3-(trimethylsilyl)prop-2-yn-1-yl)phosphonate (Bn-**15**)

NaHMDS (2 M in THF, 1.0 mL, 2.0 mmol, 1.0 equiv) was added to dibenzyl phosphite (0.44 mL, 2.0 mmol, 1.0 equiv) in anhydrous THF (3 mL) at −10 °C and the mixture was stirred for 15 min. (3-bromo-prop-1-ynyl)-trimethylsilane (0.33 mL, 2.0 mmol, 1.0 equiv) was added and stirring was continued for 1 h. Then, the reaction was quenched by addition of water (10 mL) and the aqueous layer was extracted with EtOAc (2 × 20 mL). The combined organic layers were washed with aqueous HCl (2 M, 30 mL) and water (30 mL). After drying over Na_2_SO_4_, purification by flash column chromatography (pentane/EtOAc = 7:3) gave the desired phosphonate Bn-**15** as a colorless oil (490 mg, 1.3 mmol, 66%).

TLC: *R*_f_ = 0.23 (pentane/EtOAc = 8:2). ^**1**^H NMR (CDCl_3_, 400 MHz): δ = 7.39–7.28 (m, 10 H), 5.18–5.03 (m, 4 H), 2.84 (s, 1 H), 2.79 (s, 1 H), 0.13 (s, 9 H) ppm. ^**13**^C{^1^H} NMR (CDCl_3_, 101 MHz): 136.1 (d, *J* = 6.2 Hz), 128.7, 128.6, 127.9, 95.5 (d, *J* = 14.3 Hz), 88.4 (d, *J* = 8.8 Hz), 68.5 (d, *J* = 6.6 Hz), 19.8 (d, *J* = 144.8 Hz), – 0.1 (d, *J* = 1.3 Hz) ppm. ^31^P NMR (CDCl_3_, 162 MHz): 21.8 (s) ppm. HRMS (ESI) *m*/*z* for [M + Na]^+^ calculated for C_20_H_25_O_3_PSiNa 395.1203; found 395.1199.

#### (4S)-4-(3-(trimethylsilyl)prop-2-yn-1-yl)dinaphtho[2,1-d:1′,2′-f][1,3,2]dioxaphosphepine 4-oxide (BINOL-**15**)

To *rac*-BINOL (500 mg, 1.7 mmol, 1.0 equiv) in anhydrous CH_2_Cl_2_ (12 mL) at 0 °C was added Et_3_N (0.63 mL, 4.5 mmol, 2.6 equiv) and methyl dichlorophosphite (0.20 mL, 2.1 mmol, 1.2 equiv) successively. The mixture was stirred for 12 h while warming to r.t.. Et_2_O (50 mL) was added and the resulting suspension was filtered over Celite with Et_2_O washings. Evaporation *in vacuo* yielded the crude phosphite that was taken to the next step without further purification.

The crude phosphite was dissolved in 3-bromo-1-(trimethylsilyl)-1-propyne (2.5 mL) and stirred at 160 °C by heating with a sand bath for 2 h. After cooling to r.t., direct purification by flash column chromatography (pentane/Et_2_O = 1:1) gave the desired phosphonate BINOL-**15** as an off-white foam (170 mg, 0.4 mmol, 22% over two steps).

TLC: *R*_f_ = 0.17 (pentane/Et_2_O = 6:4). ^1^H NMR (CDCl_3_, 400 MHz): δ = 8.02 (dd, *J* = 8.9, 4.4 Hz, 2 H), 7.93 (d, *J* = 8.2 Hz, 2 H), 7.59 (dd, *J* = 8.9, 0.8 Hz, 1 H), 7.55 (d, *J* = 8.9 Hz, 1 H), 7.50–7.43 (m, 2 H), 7.35 (d, *J* = 8.3 Hz, 1 H), 7.33–7.26 (m, 3 H), 3.19–2.97 (m, 2 H), 0.01 (s, 9 H) ppm. ^13^C{^1^H} NMR (CDCl_3_, 101 MHz): 147.8 (d, *J* = 10.5 Hz), 145.8 (d, *J* = 10.0 Hz), 132.6 (d, *J* = 1.3 Hz), 132.5 (d, *J* = 1.6 Hz), 132.0 (d, *J* = 1.4 Hz), 131.8 (d, *J* = 1.2 Hz), 131.5 (d, *J* = 1.5 Hz), 131.3 (d, *J* = 0.7 Hz), 128.7 (d, *J* = 0.6 Hz), 128.5 (d, *J* = 0.5 Hz), 127.4, 127.1, 127.0, 126.9, 126.0, 126.0 (d, *J* = 0.5 Hz), 121.9 (d, *J* = 2.6 Hz), 121.6 (d, *J* = 2.1 Hz), 121.1 (d, *J* = 2.1 Hz), 120.4 (d, *J* = 3.7 Hz), 93.0 (d, *J* = 14.4 Hz), 90.0 (d, *J* = 9.2 Hz), 17.8 (d, *J* = 141.0 Hz), −0.30 (d, *J* = 1.2 Hz) ppm. ^**31**^P NMR (CDCl_3_, 162 MHz): 30.0 (s) ppm. HRMS (ESI) *m*/*z* for [M + H]^+^ calculated for C_26_H_24_O_3_PSi 443.1227; found 443.1228.

#### Dimethyl (R)-(6-((*tert*-butyldiphenylsilyl)oxy)-4-methylhex-2-yn-1-yl)phosphonate (**19**)

PPh_3_ (1.79 g, 6.8 mmol, 1.0 equiv) was added to propargyl alcohol **14** (2.50 g, 6.8 mmol, 1.0 equiv) and CBr_4_ (2.26 g, 6.8 mmol, 1.0 equiv) in anhydrous CH_2_Cl_2_ (50 mL) at 0 °C. After stirring for 10 min at the same temperature, additional 3 × 0.1 equiv of PPh_3_ were added every 5 min. The reaction mixture was diluted with pentane (100 mL) and filtered over a small plug of SiO_2_ with the help of pentane/Et_2_O (98:2). Drying the filtrate *in vacuo* gave the crude propargyl bromide that was taken to the next step without further purification.

The crude propargyl bromide was refluxed in P(OMe)_3_ (15 mL) by heating with a sand bath for 12 h. Afterward, the excess of P(OMe)_3_ was removed by rotary evaporation at 70 °C. The residue was purified by flash column chromatography (pentane/EtOAc = 4:6) to yield the desired phosphonate **19** as a colorless oil (2.85 g, 6.2 mmol, 91% over two steps).

TLC: *R*_f_ = 0.43 (pentane/EtOAc = 4:6). ^**1**^H NMR (CDCl_3_, 400 MHz): δ = 7.69–7.61 (m, 4 H), 7.44–7.32 (m, 6 H), 3.82–3.69 (m, 8 H), 2.76–2.63 (m, 1 H), 2.72 (d, *J* = 2.0 Hz, 1 H), 2.67 (d, *J* = 2.2 Hz, 1 H), 1.69–1.60 (m, 2 H), 1.13 (d, *J* = 6.8 Hz, 3 H), 1.02 (s, 9 H) ppm. ^13^C{^1^H} NMR (CDCl_3_, 101 MHz): 135.70*, 135.66*, 134.1*, 134.0*, 129.70*, 129.69*, 127.74*, 127.73*, 87.5 (d, *J* = 10.2 Hz), 69.4 (d, *J* = 14.6 Hz), 53.6 (d, *J* = 6.8 Hz), 39.7 (d, *J* = 2.8 Hz), 26.9, 22.6 (d, *J* = 2.9 Hz), 21.0 (d, *J* = 3.1 Hz), 19.3, 17.2 (d, *J* = 146.5 Hz) ppm. ^31^P NMR (CDCl_3_, 162 MHz): 25.0 (s) ppm. HRMS (ESI) *m*/*z* for [M + Na]^+^ calculated for C_25_H_35_O_4_PSiNa 481.1934; found 481.1933. [α]_D_^20^ = −12 (*c* 0.05, CHCl_3_).

*1:1 splitted ^13^C NMR signals are observed for aromatic carbons in the TBDPS substituent. This indicates hindered rotation of the silyl ether, leading to rotamers.

## Data Availability

The data underlying this study are available in the published article and its Supporting Information.

## Supporting Information Available

^1^H and ^13^C{1H} NMR spectra (PDF)
