# Supplementary material for: Stereoselective Synthesis of (R)-all-trans-13,14-Dihydroretinol and -Retinoic Acid
Source: J Org Chem. 2025 Feb 20;90(9):3512–8. doi: 10.1021/acs.joc.4c03173 (PMC11894646; doi:10.1021/acs.joc.4c03173)
Supplement: Supplementary file 1 — jo4c03173_si_001.pdf [file jo4c03173_si_001.pdf]

Supporting Information for

Stereoselective Synthesis of (*R*)-All-*trans*-13,14-Dihydroretinol and -Retinoic Acid

Paul Wienecke*, Adriaan J. Minnaard*

Stratingh Institute for Chemistry, University of Groningen

Nijenborgh 7, 9747 AG, Groningen (The Netherlands)

*E-mail: p.wienecke@rug.nl, a.j.minnaard@rug.nl

Table of contents

NMR spectra S2

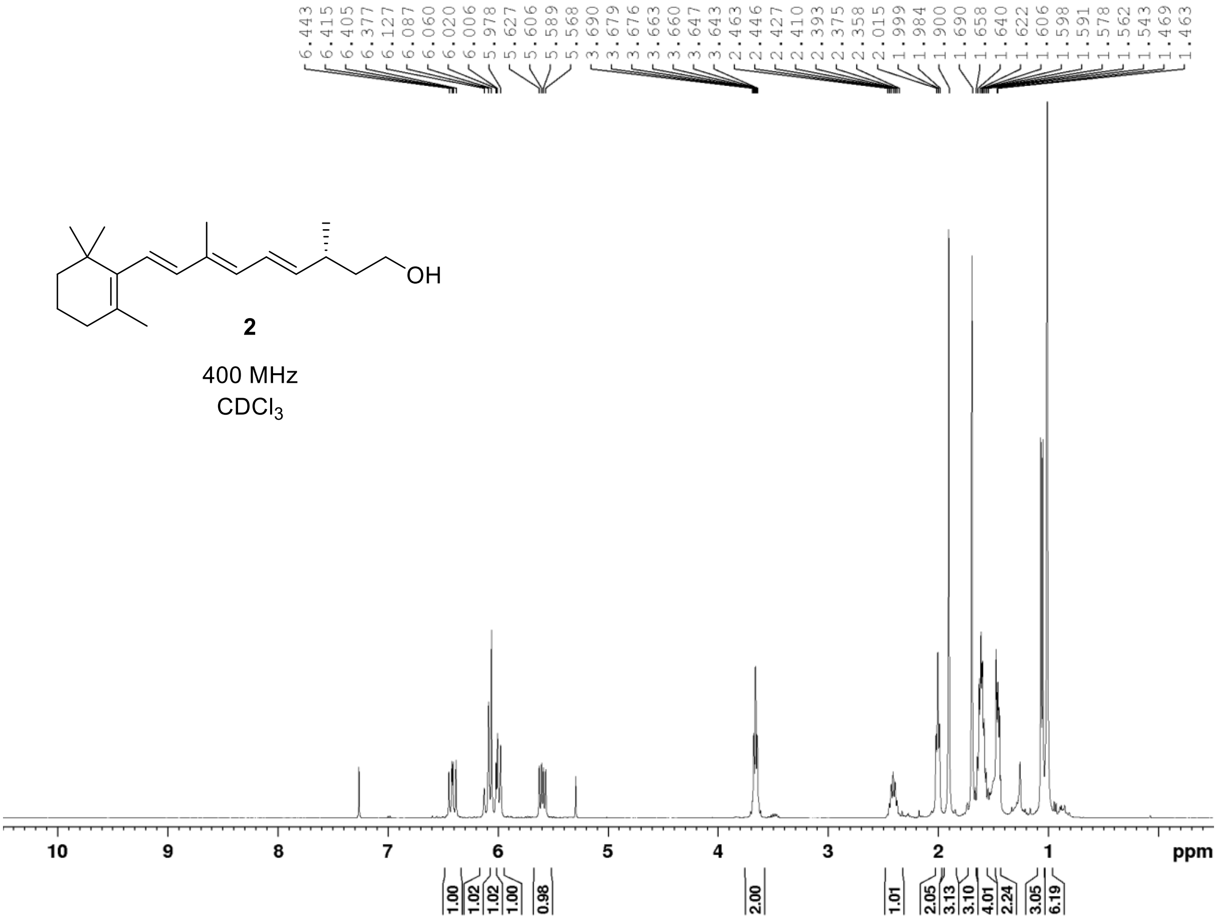

Figure S1: ^{1}H-NMR spectrum of compound **2**.

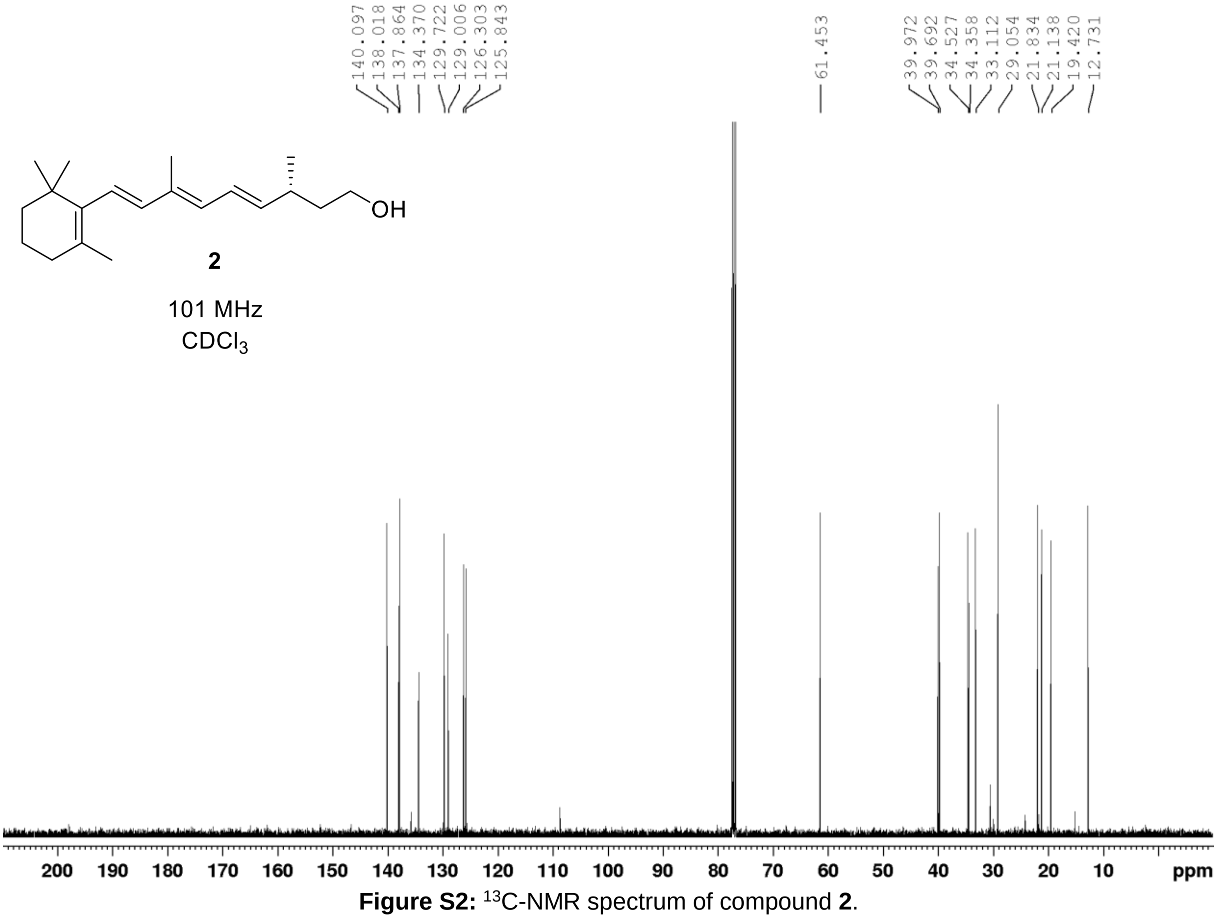

Figure S2: ^{13}C-NMR spectrum of compound **2**.

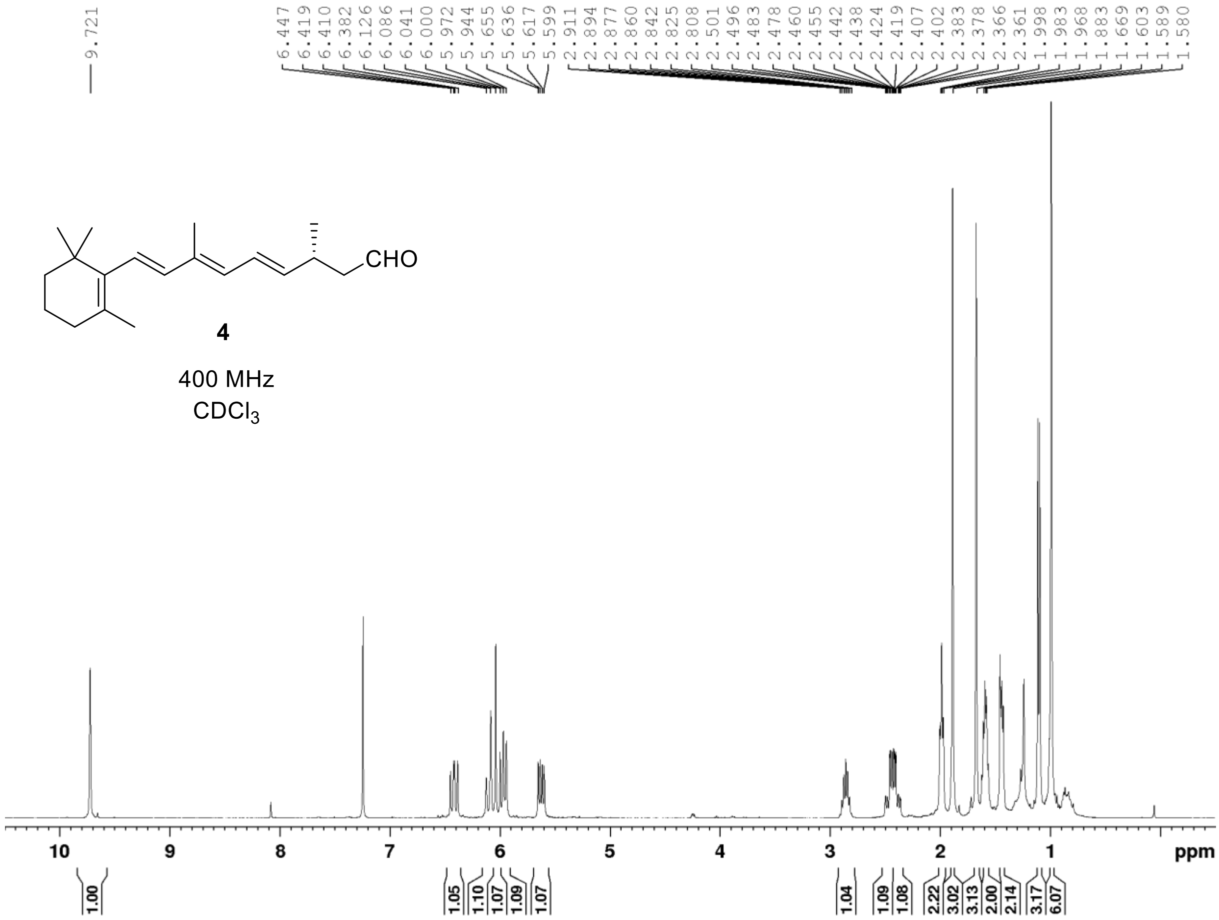

Figure S3: ^{1}H-NMR spectrum of compound **4**.

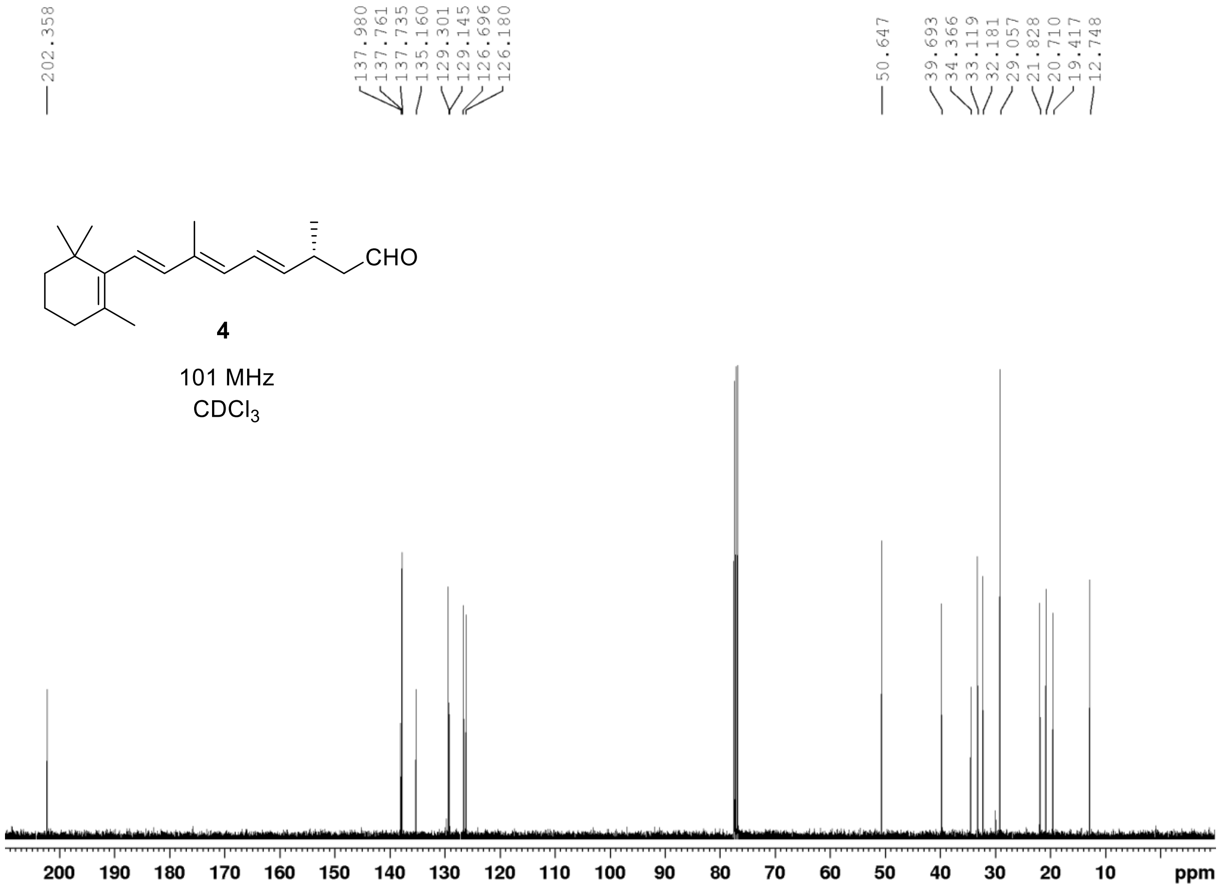

Figure S4: ^{13}C-NMR spectrum of compound **4**.

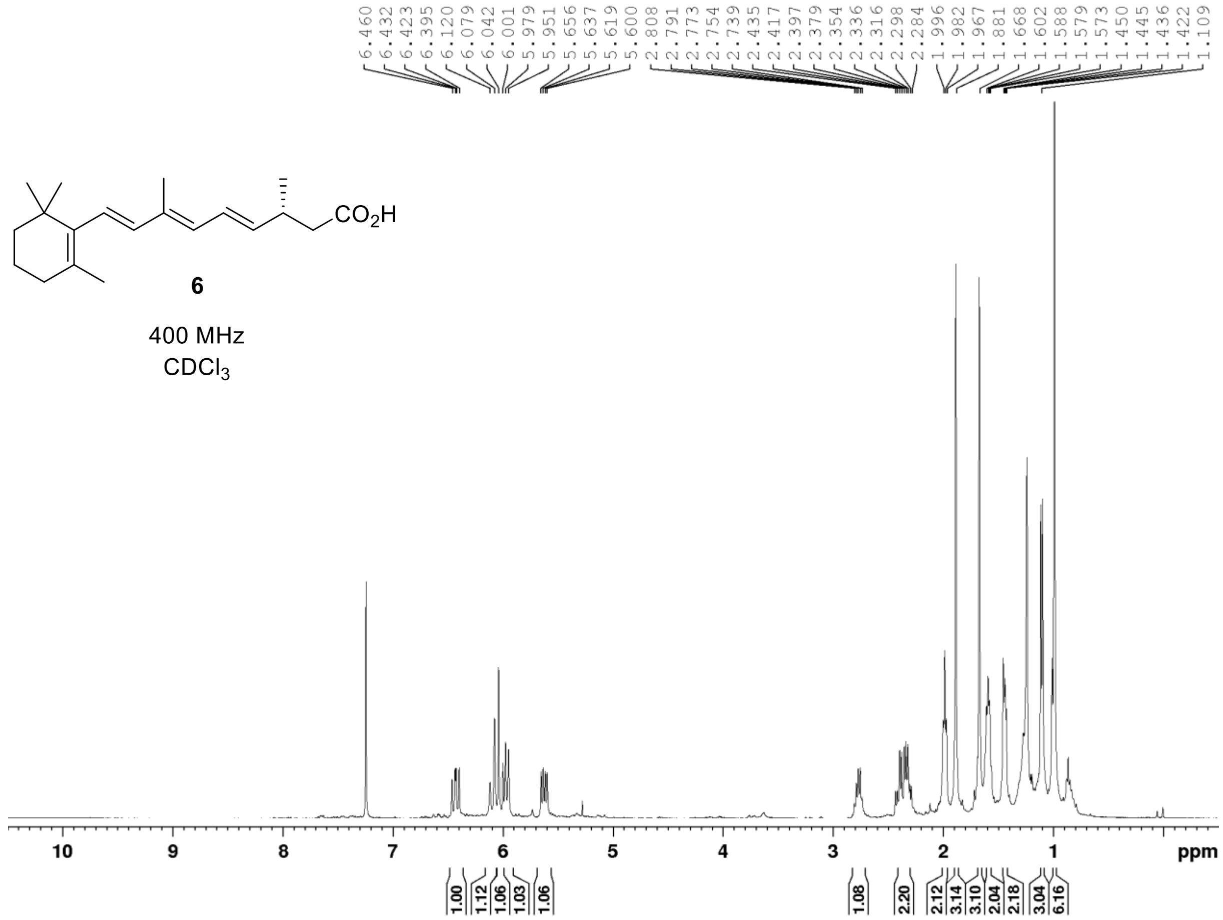

Figure S5: ^{1}H-NMR spectrum of compound **6**.

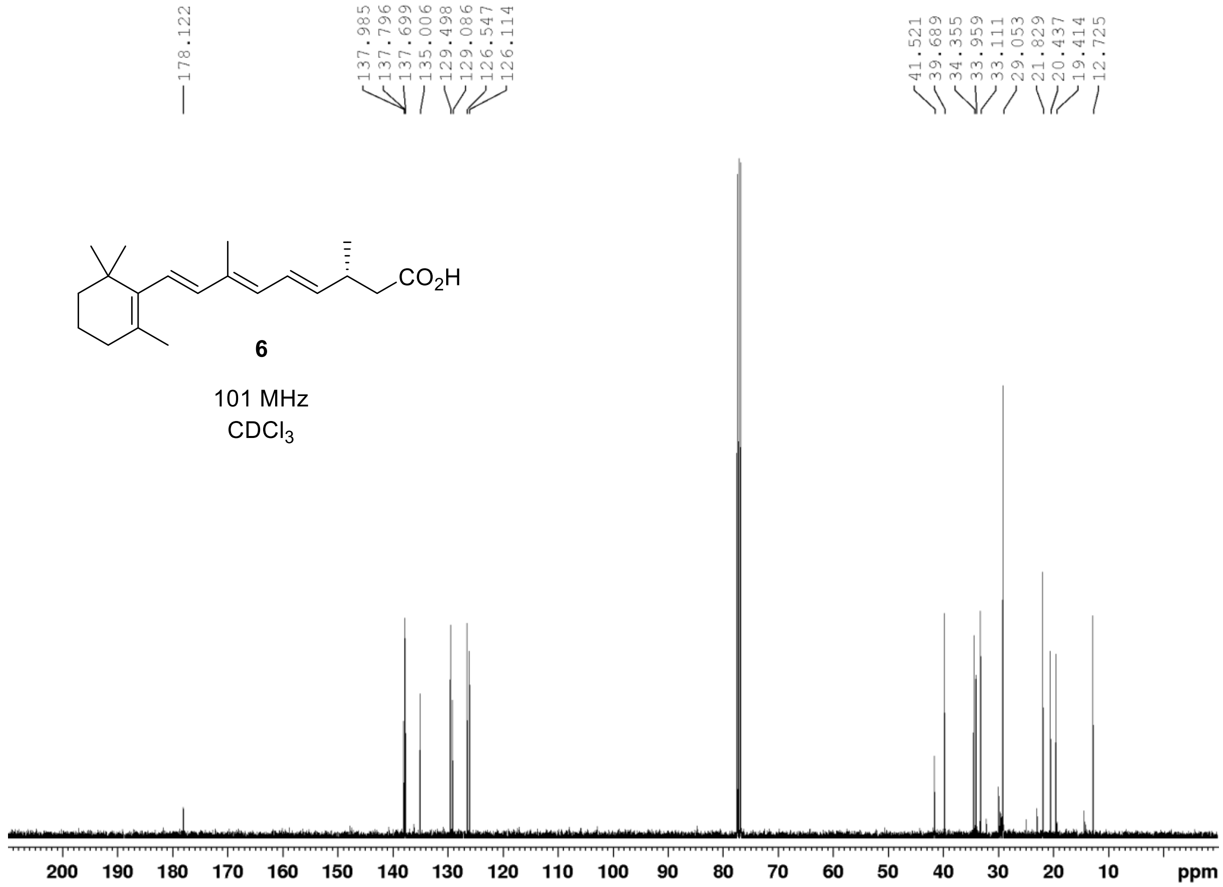

Figure S6: ^{13}C-NMR spectrum of compound **6**.

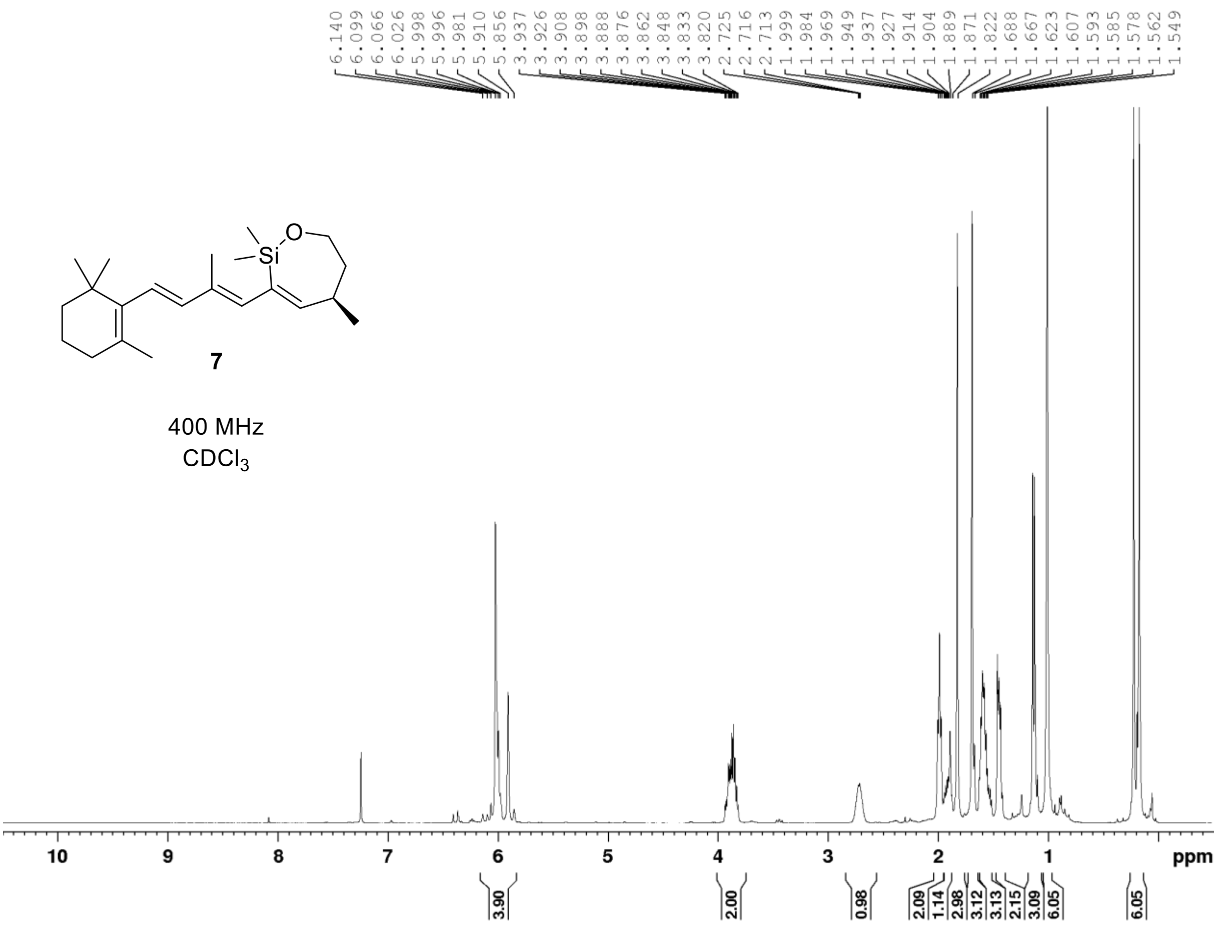

Figure S7: ^{1}H-NMR spectrum of compound **7**.

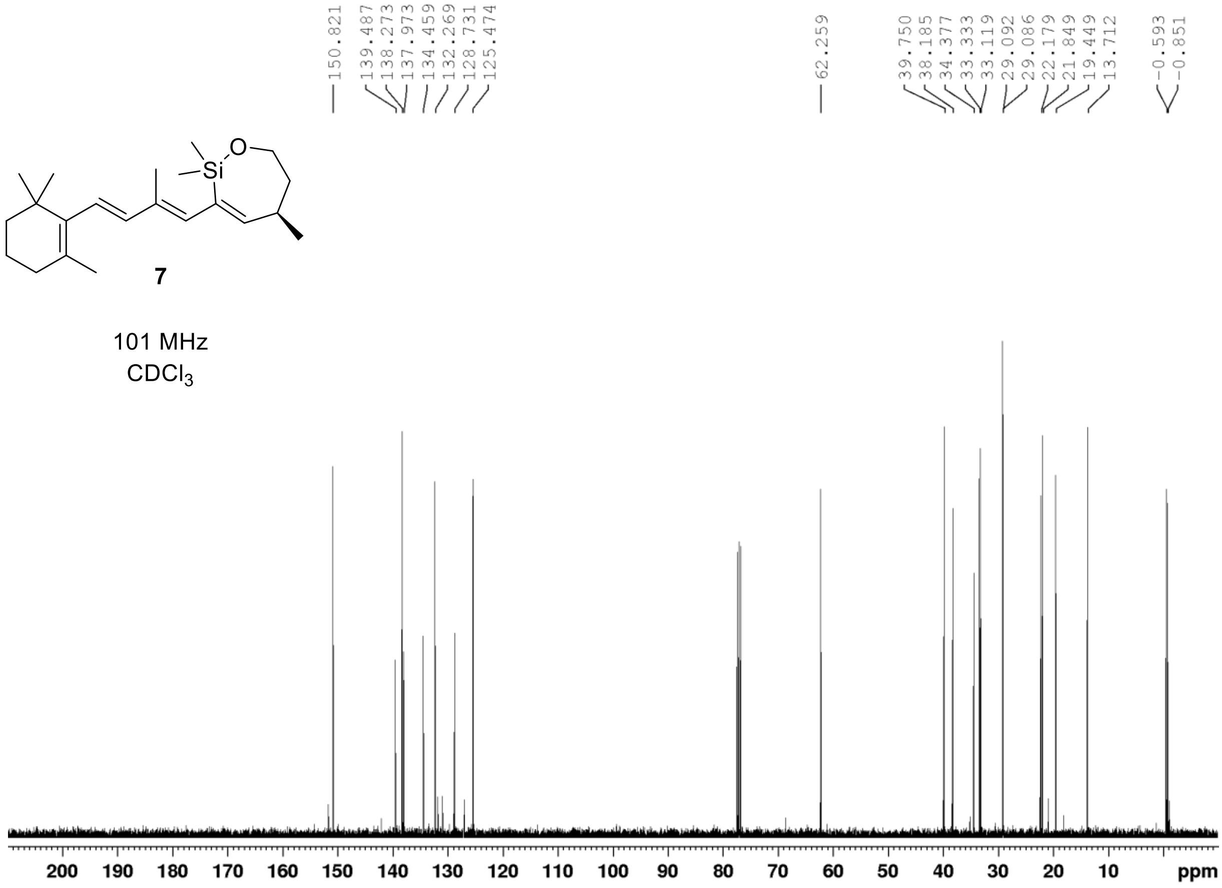

Figure S8: ^{13}C-NMR spectrum of compound **7**.

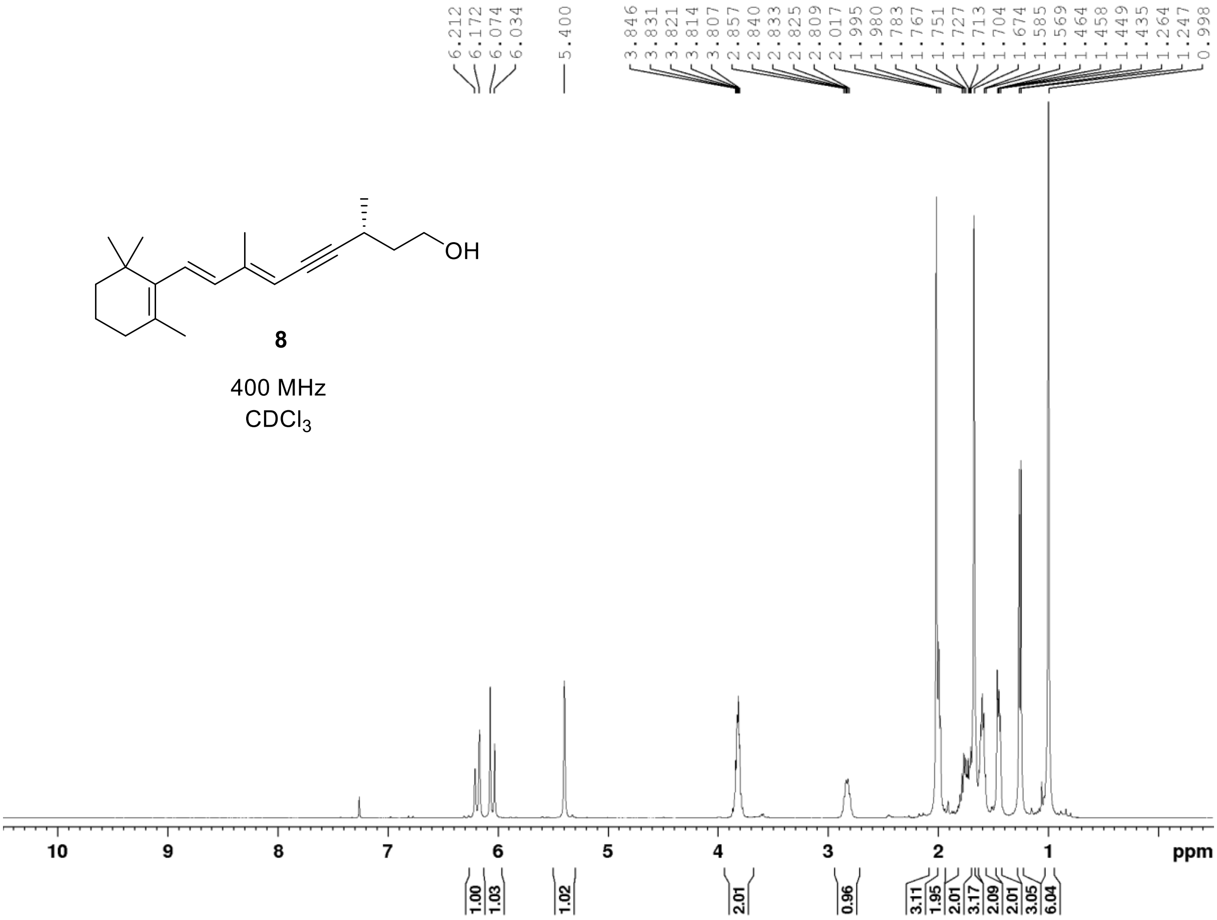

Figure S9: ^{1}H-NMR spectrum of compound **8**.

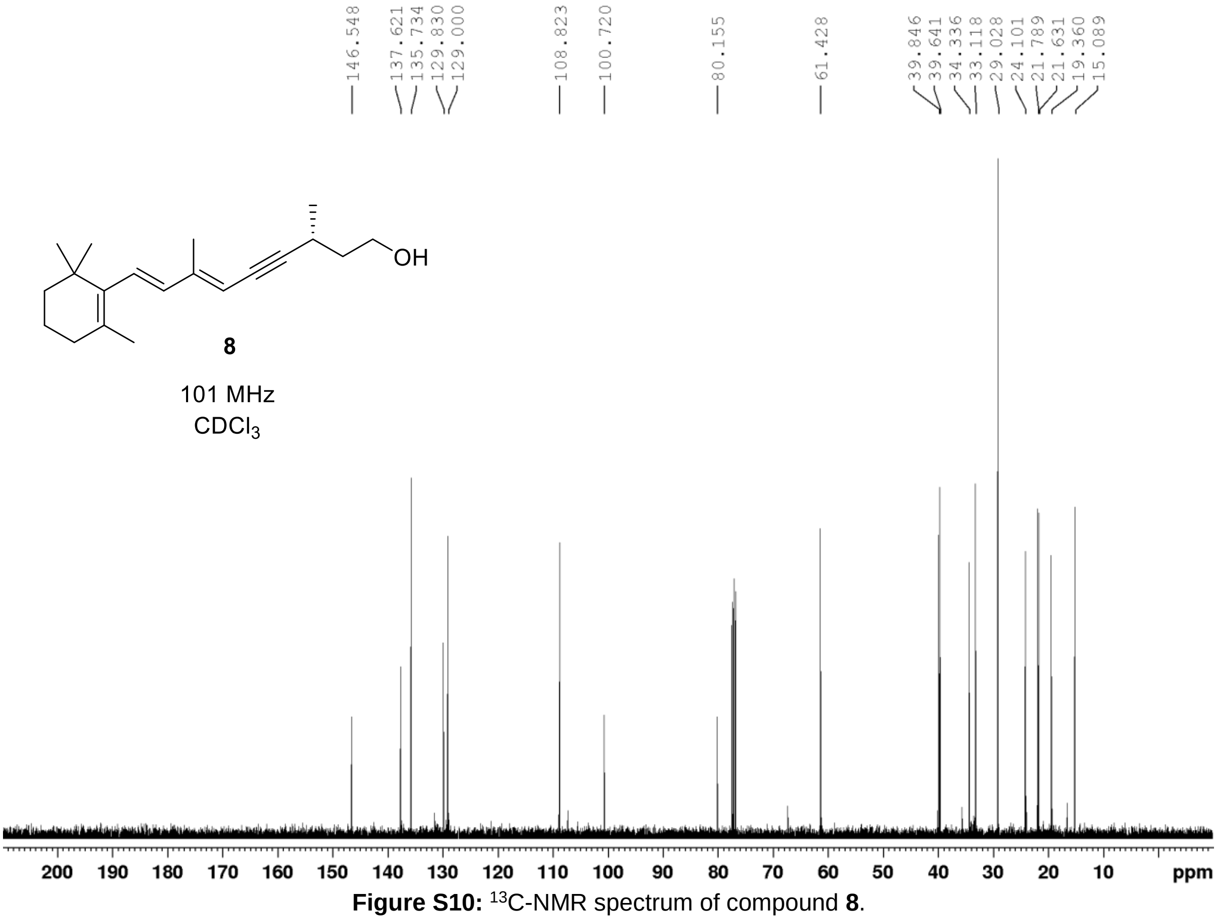

Figure S10: ^{13}C-NMR spectrum of compound **8**.

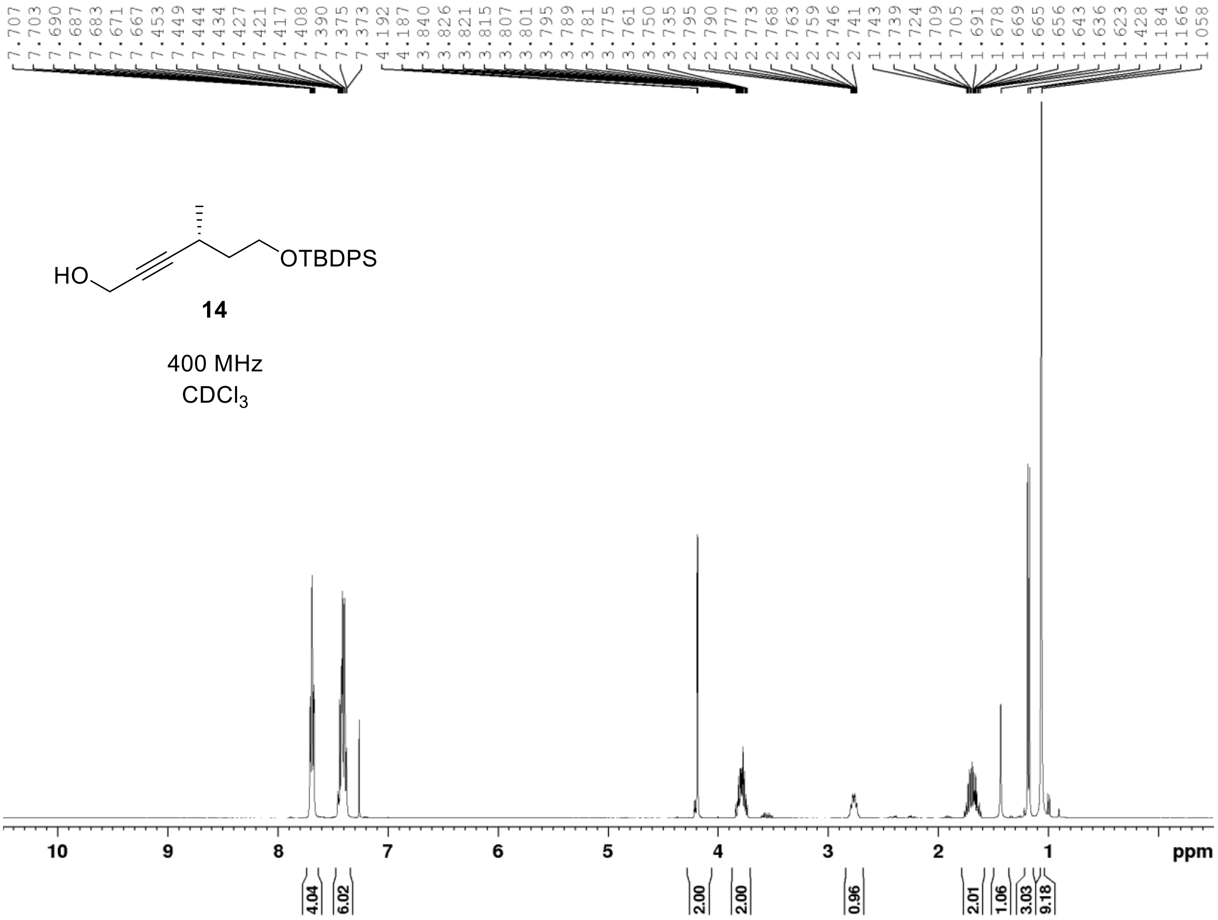

Figure S11: ^{1}H-NMR spectrum of compound **14**.

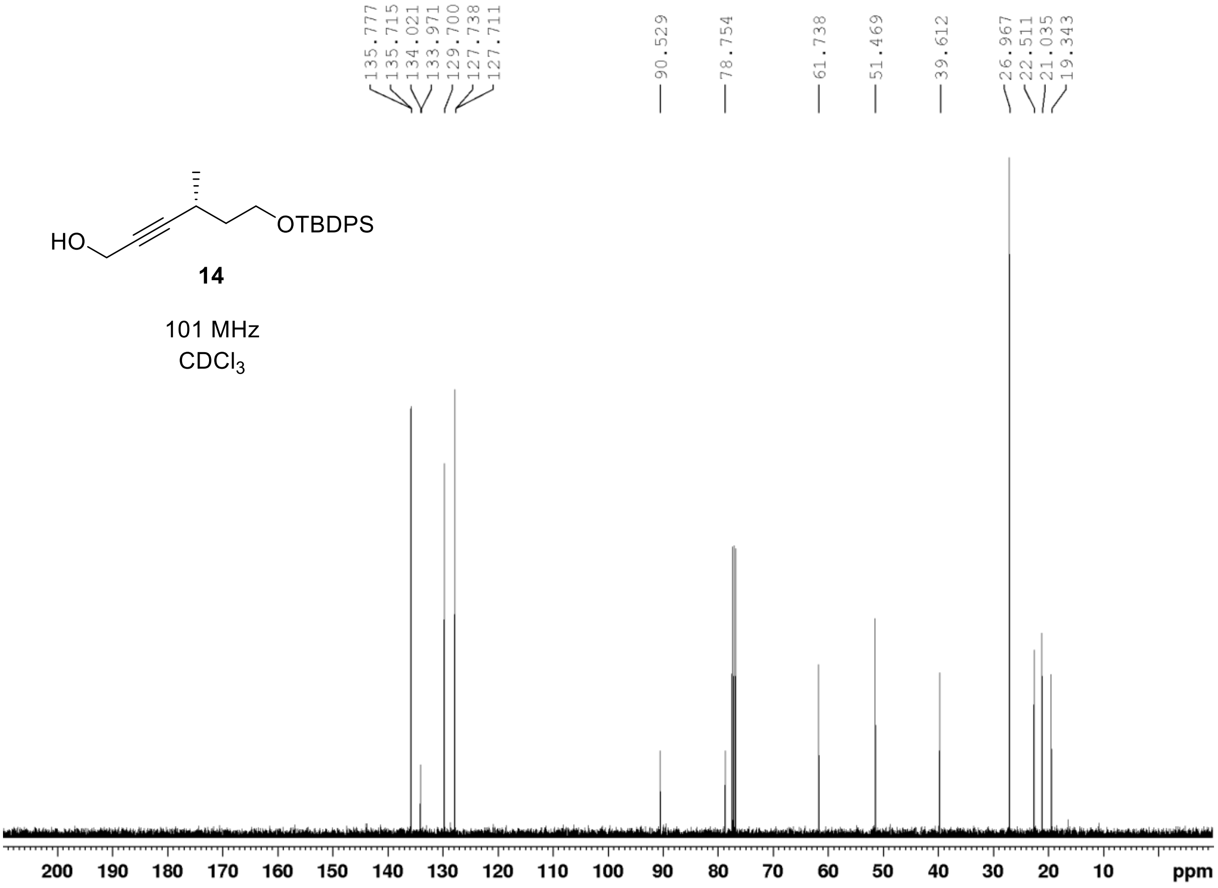

Figure S12: ^{13}C-NMR spectrum of compound **14**.

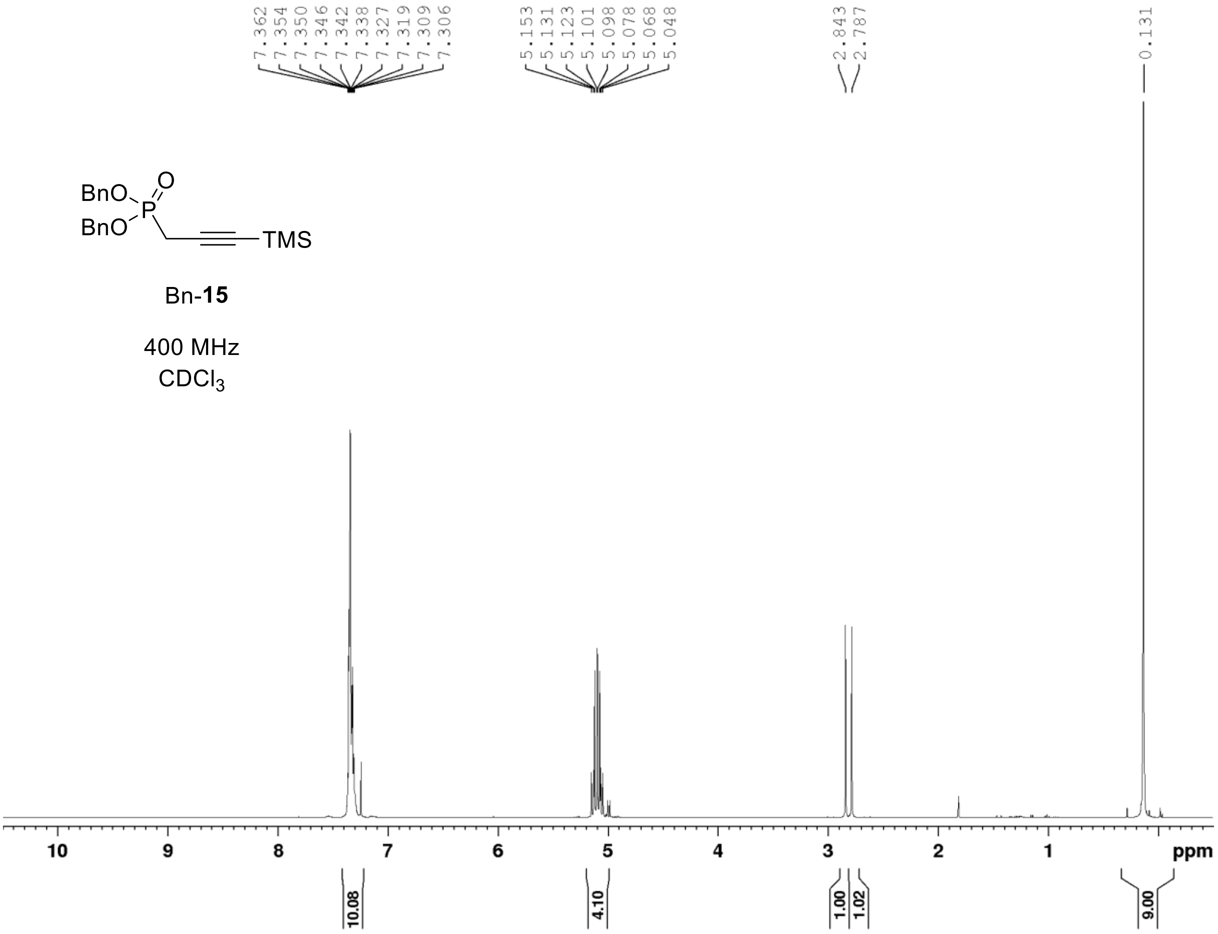

Figure S13: ^{1}H-NMR spectrum of compound Bn-**15**.

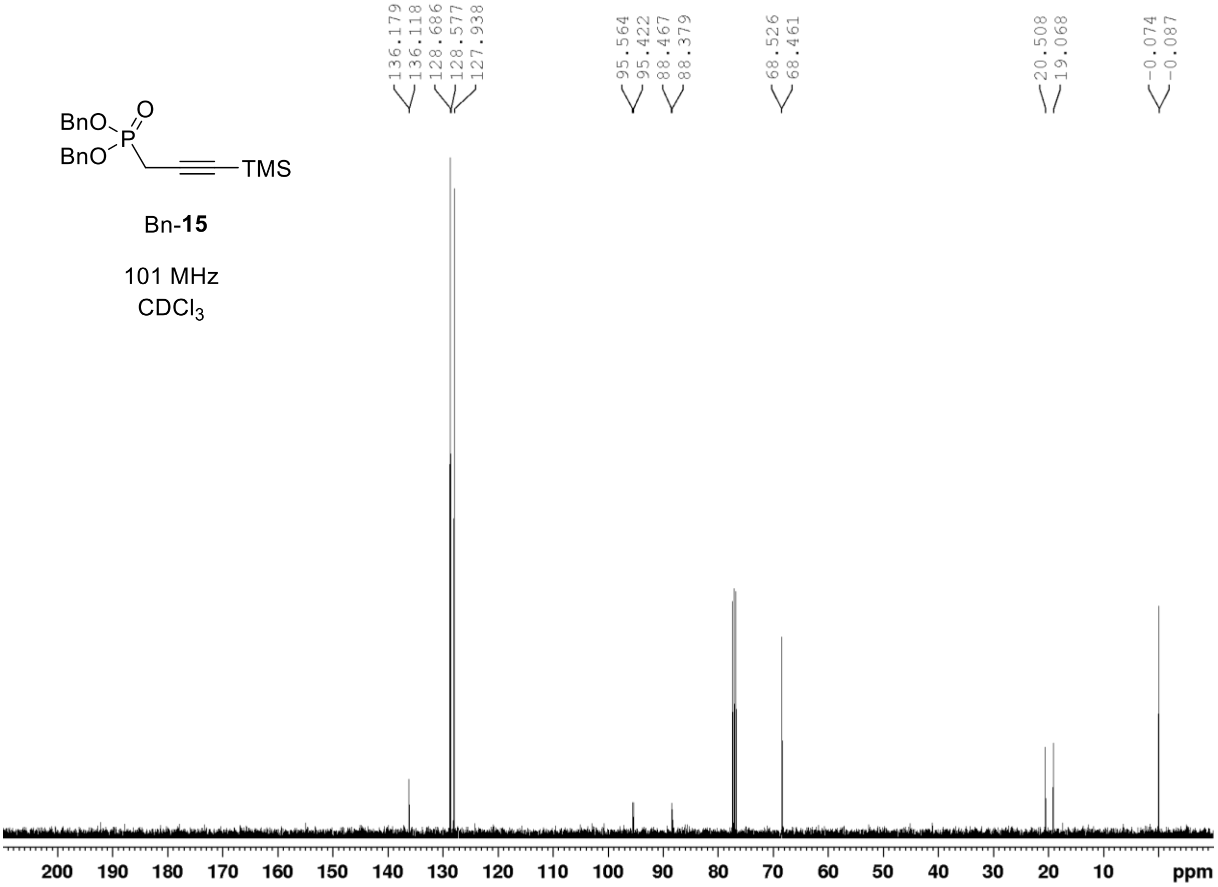

Figure S14: ^{13}C-NMR spectrum of compound Bn-**15**.

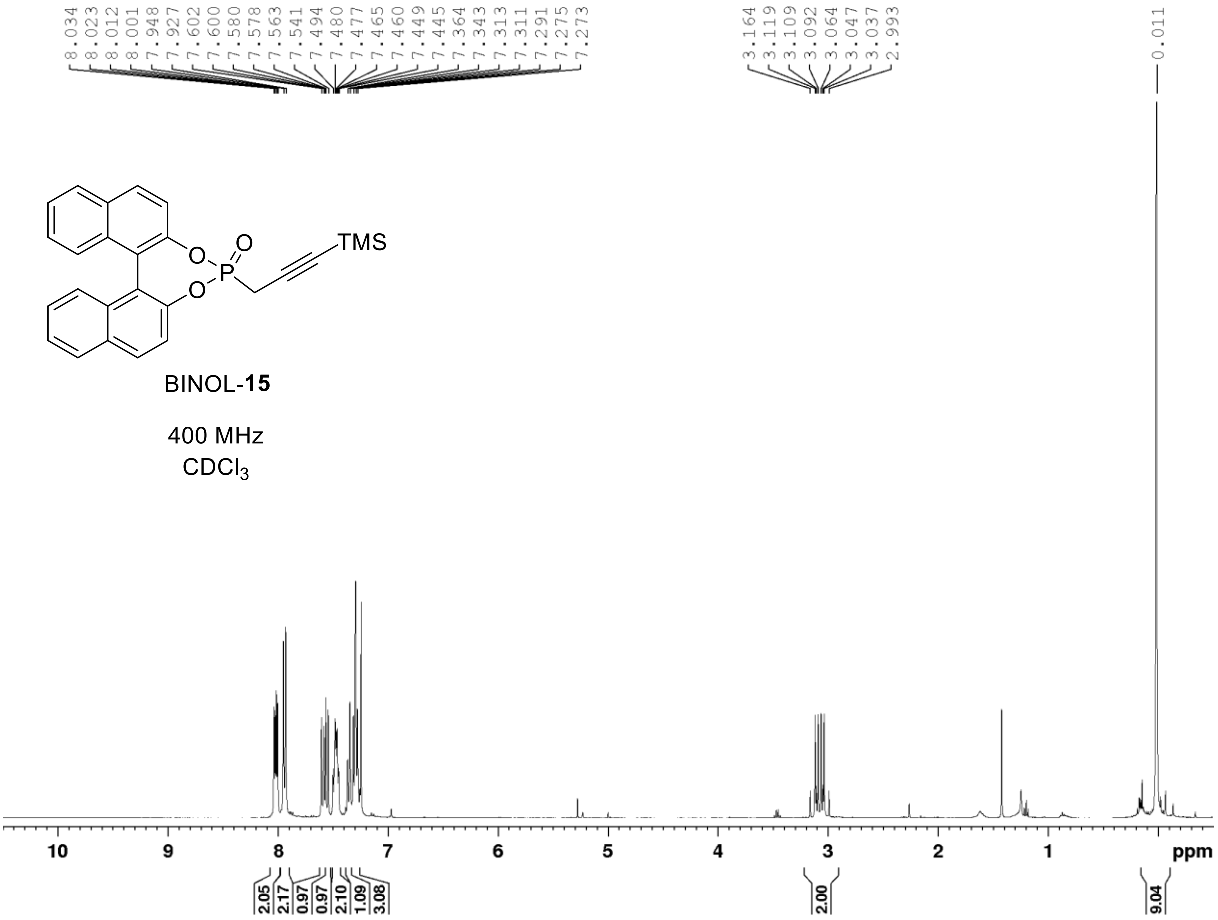

Figure S15: ^{1}H-NMR spectrum of compound BINOL-**15**.

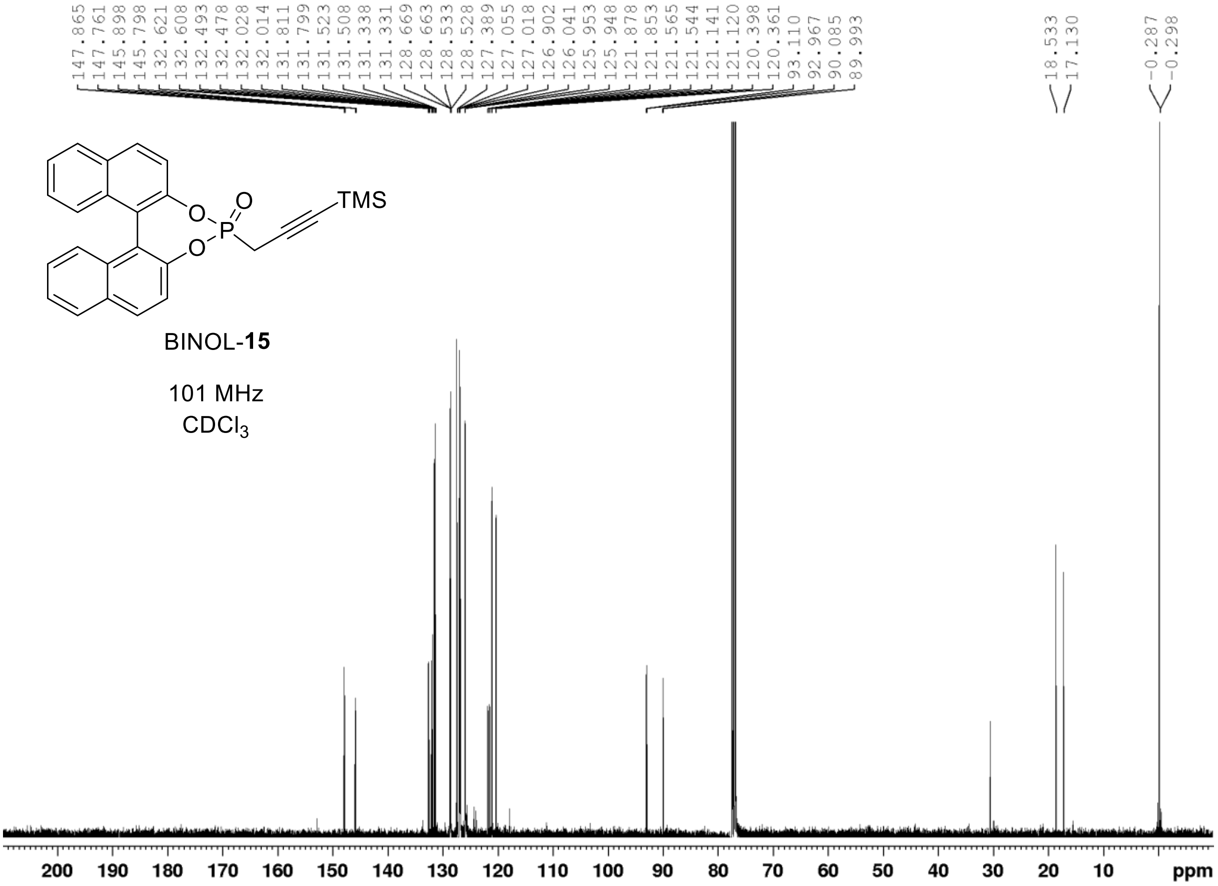

Figure S16: ^{13}C-NMR spectrum of compound BINOL-**15**.

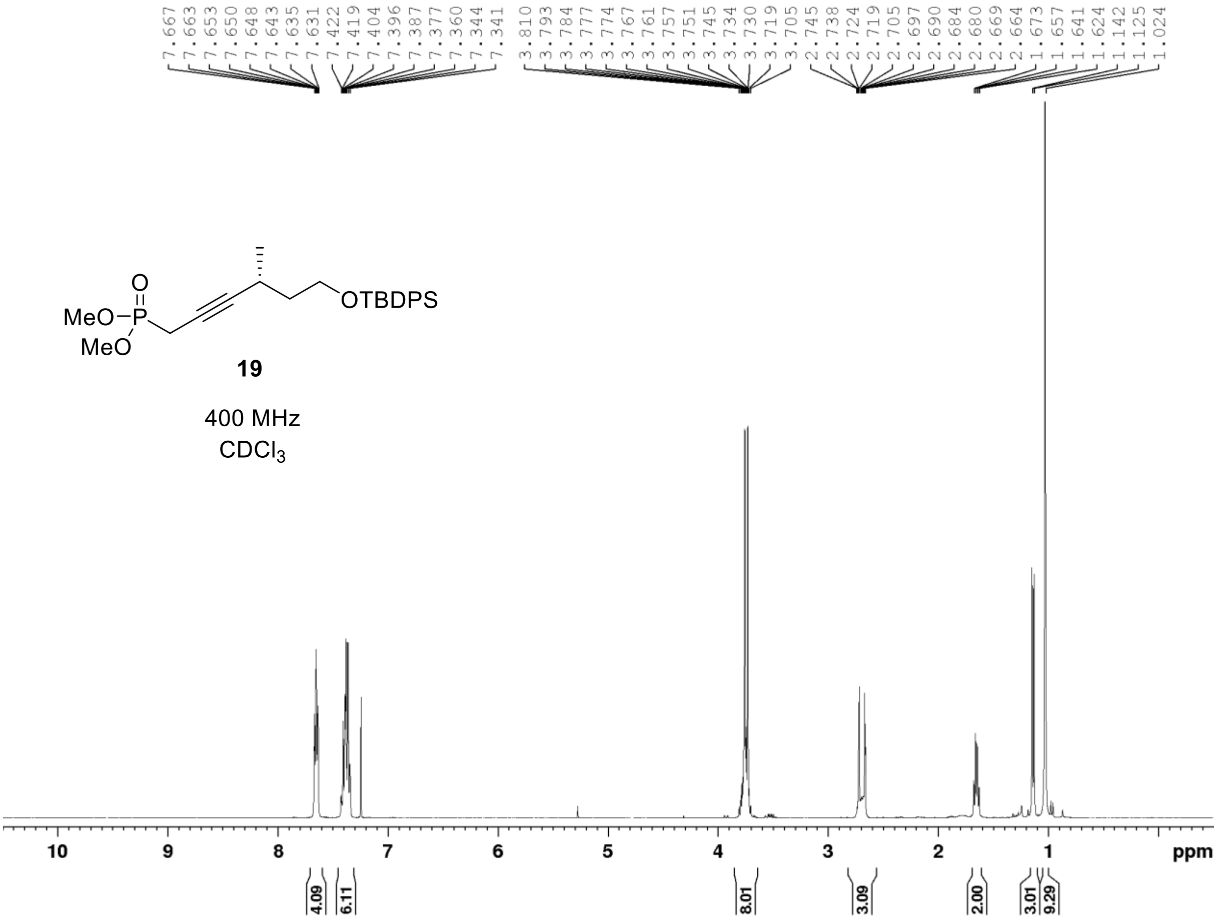

Figure S17: ^{1}H-NMR spectrum of compound **19**.

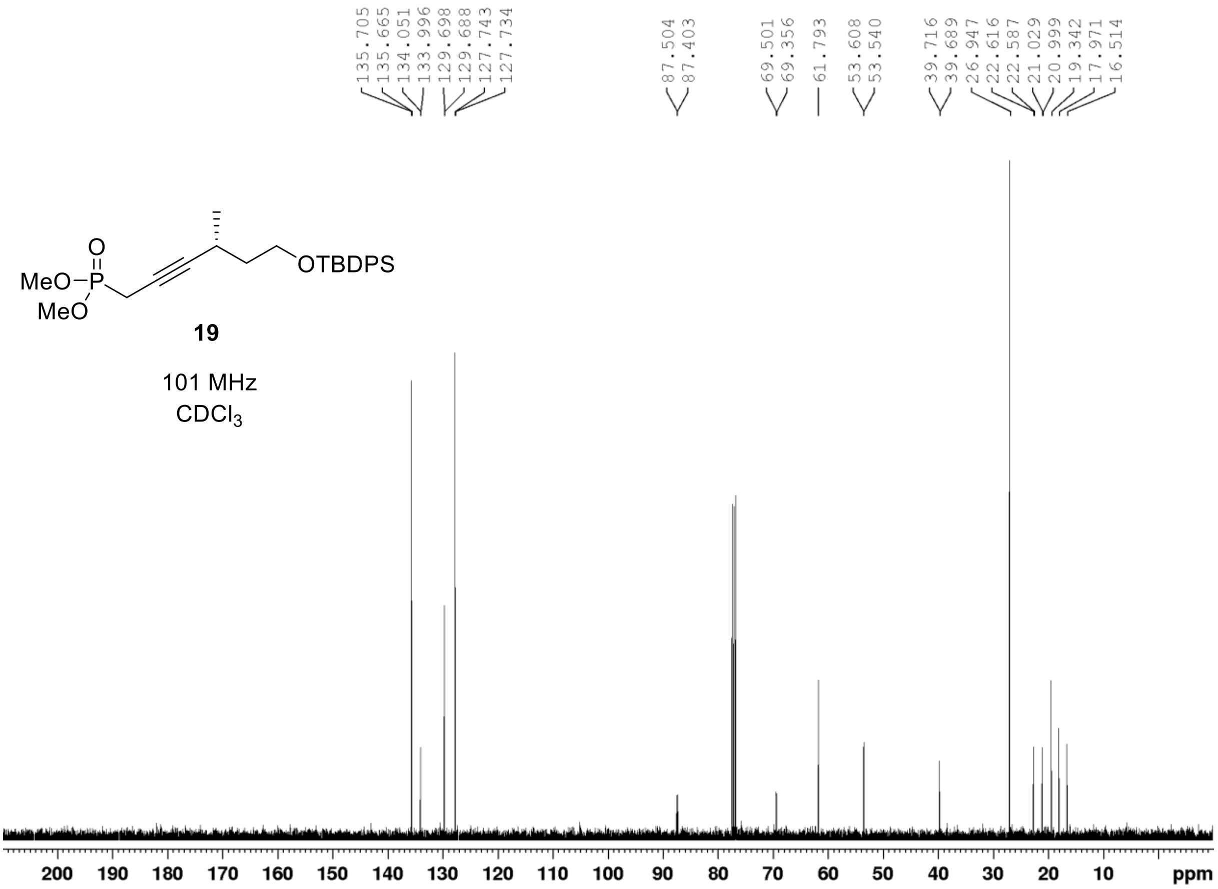

Figure S18: ^{13}C-NMR spectrum of compound **19**.
